# Evolution of Pathogen Specialisation in a Host Metapopulation: Joint Effects of Host and Pathogen Dispersal

**DOI:** 10.1371/journal.pcbi.1003633

**Published:** 2014-05-22

**Authors:** Julien Papaïx, Jeremy J. Burdon, Christian Lannou, Peter H. Thrall

**Affiliations:** 1 INRA, UMR 1290 BIOGER, Thiverval-Grignon, France; 2 INRA, UR 341 MIA, Jouy-en-Josas, France; 3 CSIRO Plant Industry, Canberra, Australia; CEFE, UMR 5175, France

## Abstract

Metapopulation processes are important determinants of epidemiological and evolutionary dynamics in host-pathogen systems, and are therefore central to explaining observed patterns of disease or genetic diversity. In particular, the spatial scale of interactions between pathogens and their hosts is of primary importance because migration rates of one species can affect both spatial and temporal heterogeneity of selection on the other. In this study we developed a stochastic and discrete time simulation model to specifically examine the joint effects of host and pathogen dispersal on the evolution of pathogen specialisation in a spatially explicit metapopulation. We consider a plant-pathogen system in which the host metapopulation is composed of two plant genotypes. The pathogen is dispersed by air-borne spores on the host metapopulation. The pathogen population is characterised by a single life-history trait under selection, the infection efficacy. We found that restricted host dispersal can lead to high amount of pathogen diversity and that the extent of pathogen specialisation varied according to the spatial scale of host-pathogen dispersal. We also discuss the role of population asynchrony in determining pathogen evolutionary outcomes.

## Author Summary

Relatively little is known about actual patterns of generalisation and specialisation in natural plant-pathogen systems largely because many fewer studies have focused on the pathogenicity structure of pathogen populations than on the resistance structure of their host populations. The spatial scale of interactions between a pathogen and its host is seen to be of prime importance in determining evolutionary trajectories of host-pathogen metapopulations because migration rates of one of the species affect the spatial and temporal heterogeneity of selection on the other. Here we develop a simulation model to specifically examine the joint effects of host and pathogen dispersal on the evolution of pathogen specialisation in a spatially explicit metapopulation. The present approach gives insights into the role of host and pathogen dispersal in driving pathogen diversity and adaptation and encourages further characterisation of the pathogenicity structure of crop and natural pathogen populations.

## Introduction

In spatially structured populations, habitat spatial heterogeneity plays a crucial role in determining the potential for species and genotypes to coexist [1], [2] and in shaping the evolution of populations and species [3], [4]. However, environments are not static and temporal fluctuations in habitat quality and distribution can also impose strong selection pressure [5]. Populations can meet this challenge by evolving or migrating [6], [7] to track high-quality environments over time [8] and in host-pathogen systems such metapopulation processes are important determinants of observed patterns of disease or genetic diversity [9], [10], [11], [12]. In particular, the spatial scale of interactions between a pathogen and its host is seen to be of prime importance in determining evolutionary trajectories of host-pathogen metapopulations [13]. Indeed, migration rates of one of the species affect the spatial and temporal heterogeneity of selection on the other [14], [15].

Some of the first models investigating the role of dispersal on host-pathogen coevolving patterns assumed a qualitative type of interaction (single locus population genetics model). The work of Gandon and colleagues [16], [17] put emphasis on local adaptation by developing a metapopulation model composed of a finite number of patches each of which could exchange propagules with its neighbouring populations. They demonstrated that asymmetry in host-pathogen dispersal can have strong effect on patterns of local adaptation. Thus when the parasite disperses more than the host, it is more likely to become locally adapted (and vice-versa). This prediction has been verified experimentally [18], [19], [20], [21] and formally qualified [17]. In a complementary way, Thrall and Burdon [22] examined the maintenance of host and pathogen genotypic diversity as a function of dispersal. They found that local dispersal for both the host and the pathogen favoured evolution of the highest number of resistance and infectivity genotypes across the metapopulation (diversity was highest when there was still some degree of among-population asynchrony).

Host-pathogen interactions are however not limited to qualitative relationships but are also largely determined by quantitative traits [23]. The role of spatial variation in host and pathogen life-history traits (components of quantitative interactions) in determining the evolutionary potential of parasitic organisms and their demographic and evolutionary histories is still poorly understood [24]. Best *et al*. [25] and Débarre *et al*. [26] modelled the evolution of host life-history traits in spatially structured host-pathogen populations. Both models assumed a lattice structure with interactions (reproduction and transmission) occurring either locally (to the nearest neighbours) or globally (randomly across the entire population). These studies underline the importance of spatial structure in affecting evolutionary outcomes. In particular, spatial structure can promote the evolution of decreased disease transmissibility but its effects on disease-related mortality depend on other aspects of life history such as the extent to which infected hosts contribute to population growth (the latter also impacts the potential for disease persistence [27]). In addition, Best *et al.* [25] studied the conditions for branching, *i.e.* when hosts undergo disruptive selection and branch into two coexisting types. They found that branching was possible in a spatial model but requires higher virulence (*i.e.* disease-related mortality) and stronger trade-offs than in a non-spatial model (*i.e.* where dispersal is homogeneous in space). However, they did not characterise the coexisting genotypes. In contrast, in a similar model but focusing on pathogen evolution, Kamo *et al.* [28] did not observe branching points for pathogen transmission and virulence. Coevolution between host and pathogen for quantitative traits was only studied by Best *et al.* [25] whose key result was that the globalisation of interactions selects for low host defence and high pathogen transmission and virulence.

Another crucial question that arises in coevolving systems is how the geographical structure of coevolution may shape spatial patterns of variation in the coevolving species [29]. Nuismer *et al.* [30] used a spatially explicit genetic model to study polymorphic clines in a one-dimensional environment. In particular, considering the more general framework of purely antagonistic interactions (including host-pathogen interactions), they found that in the absence of spatial heterogeneity in environmental conditions (which is our focus in this work) clines can only evolve when there is initial heterogeneity in allele frequencies. In addition, increases in gene flow among populations will eventually lead to the loss of spatially structured adaptation. Gavrilets and Michalakis [31] developed and analysed an island model of antagonist coevolution. Here also, when selection was homogeneous in space, the maintenance of genetic variation across time required initial differences in allele frequencies between populations and low migration rates. With increased migration, stronger selection was required. However, these two studies only considered coevolution between two alleles and neglected the important issue of genetic drift and mutation. When some spatial variations in environmental conditions are considered, one would expect that spatially heterogeneous selection together with some restrictions on migration should favour the stable maintenance of polymorphism [30], [31].

Here we develop a simulation model to specifically examine the joint effects of host and pathogen dispersal on the evolution of pathogen specialisation in a spatially explicit metapopulation. We consider a plant-pathogen system in which the host metapopulation is composed of two plant genotypes and the pathogen is dispersed by air-borne spores. We assumed that the pathogen population is characterised by a single life-history trait under selection, the infection efficacy of the pathogen on the host genotypes. We did not consider environmental spatial heterogeneity. In particular we addressed the following questions: How do the scale of dispersal and the strength of evolutionary trade-offs affect the potential for multiple pathogen genotypes to coexist? Does the level of pathogen specialisation depend on host and pathogen dispersal scales? Is there spatial heterogeneity in patterns of diversity? We first present the model and the simulation experiment. Then we study the extent of synchrony among local populations, the effect of dispersal on pathogen diversity and level of specialisation. We also analyse the sensitivity of our results to the pathogen life-history traits, to the shape of the dispersal function and to the metapopulation structure. Finally we discuss our results with an emphasis on the role of population asynchrony in determining evolutionary outcomes.

## Methods

### 2.1. Model

#### 2.1.1. Host-pathogen population dynamics

We consider a metapopulation model in which plant and pathogen populations are inter-connected via dispersal of propagules (*e.g.* seeds and spores). The model describes a polycyclic disease caused by a foliar pathogen dispersed by air-borne spores (*e.g.* rust fungus). The model is stochastic and time is considered as discrete. In population 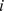 and time 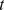, individual plants are in one of the following states (SEI model): Susceptible (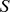), Exposed (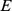) and Infectious (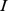).

In population 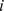, susceptible plants produce 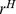 seeds (new susceptible plants) each day that migrate to other populations 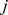 with probability 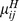. Seeds get established in population 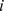 with probability 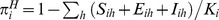, where 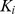 is the carrying capacity of population 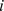. Thus, 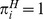 if population 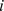 is unoccupied and 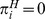 if there is no available space in population 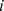. Susceptible plants die with probability 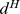.

Once infected, the plant is castrated and do not produce seeds anymore. Infected plants remain latent during 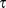 days before becoming infectious. Infectious plants produce 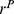 spores per day during 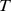 days, the infectious period, after which they are removed. Spores belong to the same genotype as their parental lesion with probability 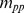. However, we assume that they can mutate from genotype 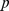 to genotype 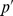 (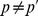) with probability 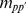. Spores migrate from population 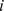 to population 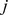 with probability 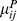.

Spores arriving on a host population contaminate a susceptible plant with probability 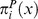, where 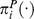 is an increasing function of 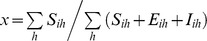, the proportion of susceptible plants in the population. The function 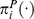 insures that, in population 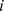, 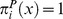 if all the plants are susceptible and 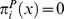 if there is no susceptible plants. Two different shapes for the density dependence function 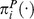 are used. First, a linear relationship is considered assuming that 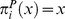. In addition, we assume that susceptible plants become less easily contaminated when the local disease level increases by using the following sigmoid function for 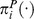:
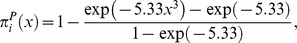
giving an inflection point for 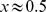 equal to 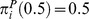. The use of a sigmoid function instead of a linear function for 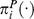 implies that the contamination of a susceptible plant is easier when the proportion of susceptible plants is higher than 50%, and harder when the proportion of susceptible plants is lower than 50%

A plant receiving a spore (contaminated plant) becomes infected with a probability 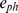, the infection efficacy of pathogen genotype 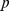 on host genotype 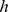. Genetic interaction between the host and the pathogen is assumed to impact only the infection efficacy 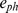 and thus only this parameter depends both on the host and pathogen genotypes.

Seeds and spores that fail to establish are removed (no seed or spore bank) which implicitly imposes a cost of dispersal. The different steps and their chronology are detailed in Appendix S1 in Text S1.

#### 2.1.2. The population network

The host and pathogen metapopulation consists of a network of 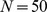 populations that covers a total proportion 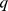 of the environment (Fig. S1 in Text S1). The centre of each population was first located randomly via a homogeneous Poisson point process inside a square region of size 1 by 1 unit. Then, population surfaces were drawn from a log-normal distribution to obtain the desired value of 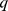. In addition, we assumed absorbing boundaries, as such scenarios best mimic metapopulations of definite size beyond which propagules are essentially lost from the system.

The probability that a spore (or respectively a seed) disperses from population 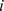 to population 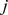, 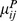 (respectively 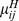), is computed from an individual dispersal function, 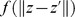, where 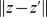 is the Euclidean distance between points 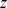 and 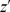. 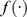 is assumed to be a Weibull function so that:
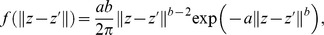
where 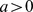 and 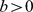. The parameter 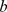 determines the shape of the dispersal function [32]: 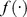 is thin for 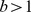, fat for 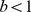 and exponential for 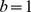. In addition the mean dispersal distances (respectively 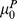 and 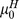 for spores and host seeds) are given by 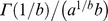, where 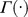 denotes the Gamma function. Dispersal probabilities are computed by integrating 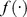 over the population surfaces using the Califlopp algorithm [33].

#### 2.1.3. Host genotypes, pathogen genotypes and evolution

The host population is composed of two resistant genotypes, 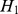 and 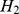, which properties do not change through time and with no mutation between them. Both host genotypes are ecologically equivalent in the absence of disease, implying no cost of resistance or susceptibility. When disease is present, the two host genotypes have different fitnesses determined by the local pathogen population (see below for more details).

The pathogen population is initially composed of one generalist genotype defined by a fixed infection efficacy on each host genotype. Specialist genotypes emerge through the balance of mutation and selection. They are defined according to the gain, in percentage of the infection efficacy of the generalist genotype, that they experience on the host to which they are adapted (their susceptible host) and the cost that they suffer on the other host (their resistant host). The gain of the generalist is by definition 0. We assume a trade-off between gain and cost such that the 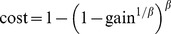. Parameter 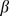 characterised the strength of the trade-off function. When 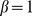, the cost is equal to the gain (linear trade-off), when 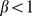, the gain is greater than the cost (weak trade-off), and when 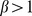, the cost is greater than the gain (strong trade-off).

Pathogen genotypes are classified according to their gain in infection efficacy on their susceptible host. Genotype 1 corresponds to the full specialist of host 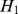 (it cannot infect the host genotype 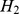) and the genotype with the highest index corresponds to the full specialist of host 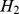 (it cannot infect the host genotype 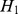). The other specialised genotypes are considered as moderate specialists since they can infect both hosts. In addition, we assume that evolution is gradual: a genotype can produce closely related mutant genotypes only (*i.e.* those with small gains or losses in 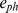).

### 2.2. Simulation experiment

Ten mean dispersal distances (in proportion of the region size) were considered for both host and pathogen by varying 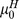 and 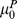 in {1.25%, 2.5%, 5%, 7.5%, 10%, 17.5%, 25%, 37.5%, 50%, 75%}.

We fixed the infection efficacy of the generalist to 0.2. In addition to the generalist we defined 10 specialists on each host (thus 20 specialists) by increasing 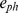 by 10%, 20%, …, 90% and 100% on their susceptible host. Finer steps for the discretisation of 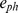 did not change the results. Two different scenarios were explored with regard to the cost that specialists suffered on their resistant host: 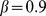 (the specialisation gain is greater than the specialisation cost) and 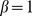 (the specialisation gain is equal to the specialisation cost). The case 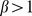 is not reported in the present study because no qualitative differences were observed relative to the case when 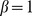: 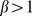 only stabilised the generalist population even more.

The probability that a spore was of the same genotype as its parental lesion was set as 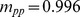, then we set 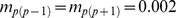. Exceptions were the two full specialist pathogen genotypes (gain  =  cost  = 100%) – these mutated toward less specialised genotypes (gain equal to 90%) with probability 0.004 to keep their overall mutation rate equal to that of other genotypes.

Sensitivity to the other parameters (spore production, 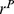; infectious period, 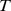; latency period 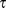; shape of the density dependence function, 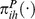; shape of the dispersal function, 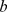 and total proportion covered by the metapopulation, 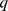) was assessed by studying the 7 case-studies detailed in Table 1.

**Table 1:** Values of parameters used in the case-studies.

Case study	Spore production ( 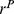 )	Infectious period ( 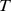 )	Latency period ( 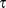 )	Density dependence ( 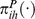 )	Dispersal function shape ( 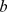 )	Metapopulation coverage ( 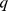 )
A	2	10	5	Sigmoid	1	5%
B	4	10	5	Sigmoid	1	5%
C	2	20	5	Sigmoid	1	5%
D	2	10	10	Sigmoid	1	5%
E	2	10	5	Linear	1	5%
F	2	10	5	Sigmoid	0.8	5%
G	2	10	5	Sigmoid	1	10%

The case-study A is considered as the reference.

The convergence was checked on a subset of simulations by computing the descriptors of the global pathogen evolutionary trajectory (see Section 2.3.2) at different times until they stabilised. Simulations were thus performed over 20,000 time steps. The system started with the two host genotypes present in all patches and with the pathogen population composed of the generalist only. For each case-study, five different metapopulations were drawn and four model replicates were performed on each of them leading to 20 replicates for each of the 1400 scenarios (10 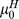 by 10 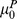 by 2 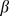 by 7 case-studies). Table 2 summarises the terms, parameters and values that we used.

**Table 2:** Parameter definition and values.

Parameter	Definition	Value
*Metapopulation*		
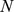	Number of populations	50
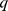	Metapopulation relative surface	5%, 10%
*Host*		
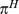	Establishment probability	Proportional to the available space
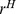	Juvenile production per day	1
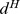	Probability of death	0.1
*Pathogen*		
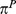	Contamination probability	Sigmoid and linear shape
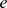	Infection efficacy	Between 0 and 0.4
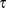	Latency period	5, 10
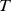	Sporulating period	10, 20
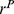	Spore production per day	2, 4
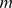	Mutation probability	0.004 to mutate toward another genotype
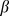	Trade-off shape	{0.9, 1}
*Dispersal*		
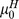 , 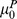	Host and pathogen mean dispersal distance	Between 1.25% and 75% of the region size
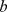	Dispersal function shape	{0.8, 1}

### 2.3. Outputs

#### 2.3.1. Spatial structure

We focus here on the metapopulation spatial structure. Based on the dynamics of the host genotype 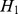 during the last 2000 time steps (eventually, taking the host genotype 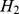 would not change the results), we first characterised synchrony among local populations by computing the mean correlation among 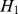 metapopulation dynamics and 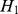 local population dynamics. An elevated mean correlation indicated that both local populations and the metapopulation underwent the same dynamics: *i.e.* the entire metapopulation was synchronised. We then assessed how the synchrony decayed with distance by estimating the spatial spline centred correlogram among 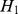 local population dynamics using the R package ncf [34]. Bootstrap confidence envelopes were constructed based on 1000 samplings with replacement among populations.

#### 2.3.2. Evolutionary trajectories

**Figure 1:**
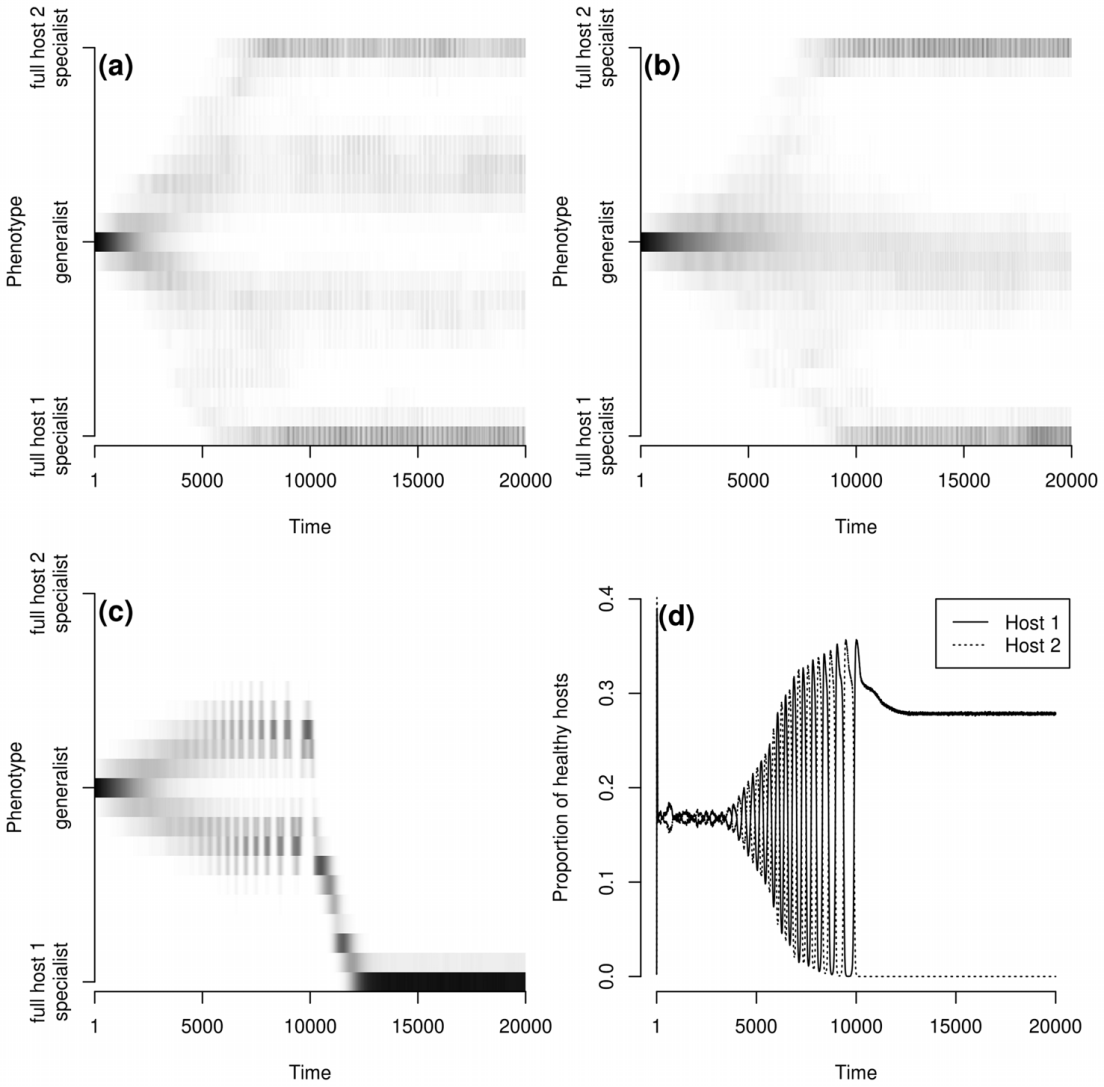
Examples of pathogen evolutionary trajectories (a, b and c) and host dynamics (d). **a** and **b**: 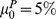, 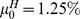; **c** and **d**: 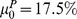, 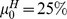. **a**, **c** and **d**: 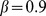; **b**: 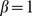. An exponential dispersal function (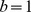) is used and the metapopulation covers 5% of the environment. The grey intensity indicates the frequency of pathogen genotypes, white: the frequency is equal to 0, black: the frequency is equal to 1.

We considered here the entire metapopulation by aggregating local evolutionary trajectories across the metapopulation. This gave patterns of pathogen evolution as presented in Fig. 1a, b and c. Based on the last 2000 time steps we determined the ‘genetic clusters’ that persisted at the end of the simulation. A ‘genetic cluster’ was defined as a group of closely related pathogen genotypes whose metapopulation size, averaged over the last 2000 time steps, was greater than 5% of the total pathogen metapopulation size (*e.g.* four genetic clusters are visible in Fig. 1a, three in Fig. 1b and one in Fig. 1c). We then characterised (i) the number of genetic clusters that persisted (the more clusters, the greater the diversity), (ii) the median of the infection efficacy over genotypes and time (hereafter referred to as 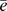) and (iii) the (2.5%, 97.5%) quantile interval of the infection efficacy over genotypes and time (hereafter referred to as the ‘efficacy range’) on the susceptible host of each genetic cluster. 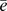 and the efficacy range describing the genetic clusters in terms of specialisation level (due to the trade-off function, the highest 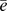 the more specialised the genetic cluster is) and homogeneity (the highest the efficacy range the less homogeneous the genetic cluster is), respectively.

The effects of the input factors on the descriptors of the pathogen evolutionary trajectory were assessed by fitting a multinomial logistic regression model (R package nnet [35]), for the number of genetic clusters, and penalized Generalised Additive Models with tensor product smooths (R package mgcv [36]), for 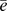 and the efficacy range, respectively.

## Results

The results presented in Sections 3.1, 3.2 and 3.3 are based on the case-study A (Table 1). The simulations issued from the other case-studies (Table 1, case-studies B to G) were used in Section 3.4 to assess the sensitivity of the outputs to the latency and infectious periods, the shape of the density dependence function, the pathogen reproduction, the 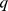 value and the shape of the dispersal function.

### 3.1. Spatial partitioning of genotypes and synchrony among populations

Here we focus on local populations and the extent to which they were synchronised to characterise the spatial structure of the metapopulation. These results were used in Section 3.2 and 3.3 to explain patterns of coexistence and specialisation.

When host and pathogen dispersed very locally, populations were largely asynchronous (Figs. S2 and S3 in Text S1). Increases in both host and pathogen mean dispersal distances resulted in an increase in the level of correlation between local and global dynamics, *i.e.* an increase in synchrony. Finally when both host and pathogen dispersed at large distances, the metapopulation was totally synchronised.

**Figure 2:**
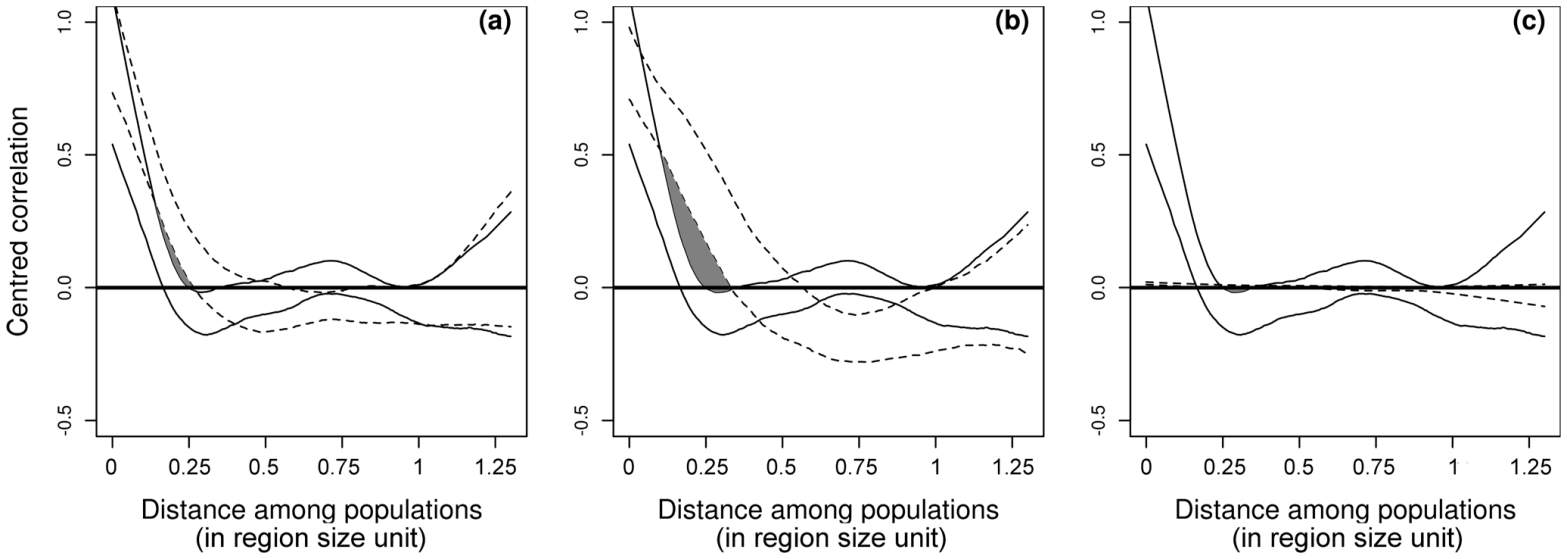
The 95% confidence envelopes for the spatial covariance function for the host genotype 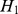 when the pathogen mean dispersal distance is 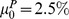 and 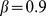. **a:** 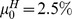 (solid line) and 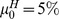
(dashed line), b: 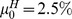 (solid line) and 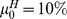 (dashed line), c: 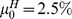 (solid line) and 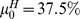 (dashed line). Significant discrepancies are highlighted in grey.

**Figure 3:**
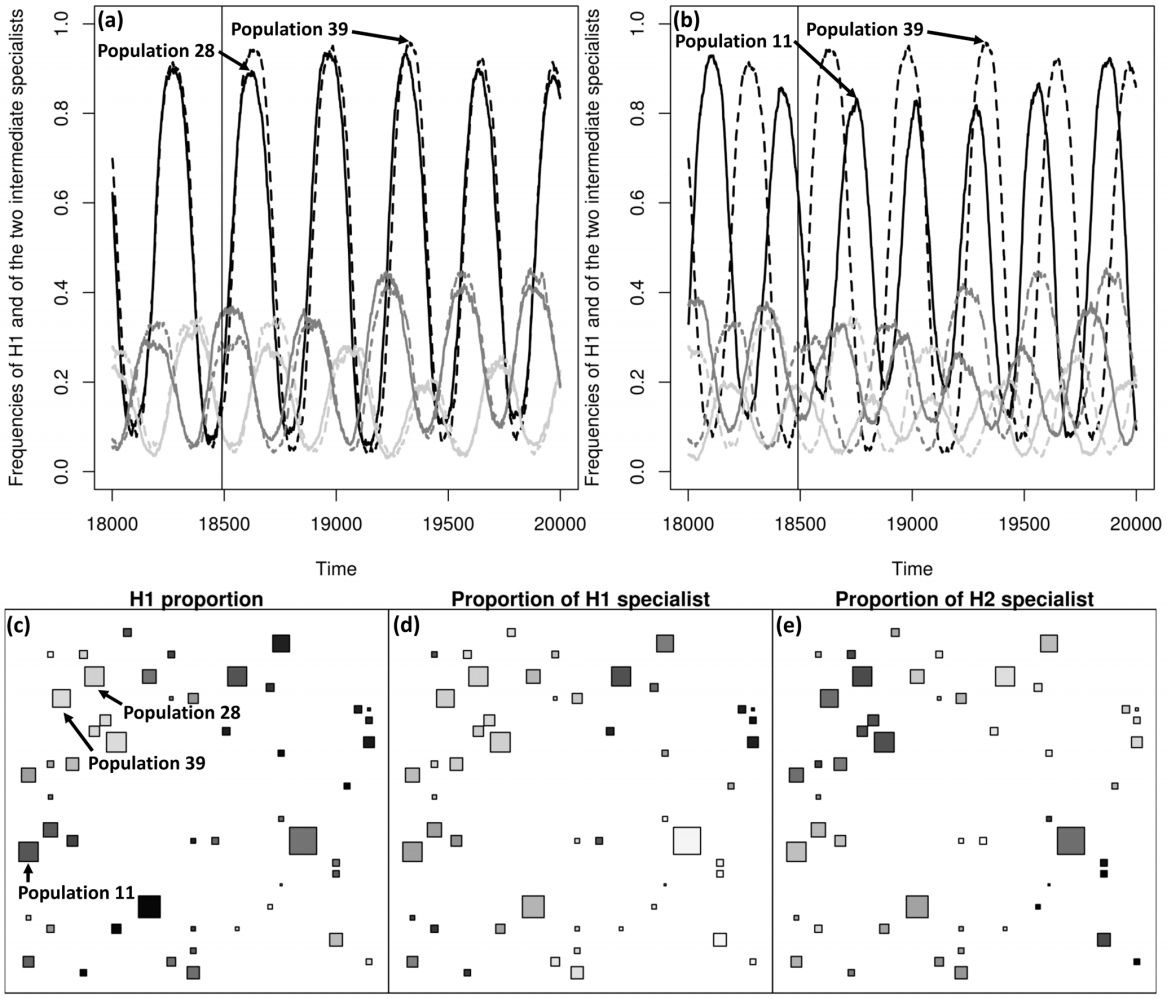
Example of population aggregates synchrony. The top row displays the dynamics of 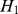 and of the two main pathogen genotypes present in the metapopulation on three different populations. The bottom row displays the population spatial structure. **a** and **b**: black, 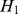 frequency; light grey, 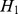 specialist frequency; dark grey, 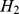 specialist frequency. **c**, **d** and **e**: 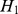, 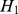 specialist and 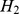 specialist proportions at the local population level at the time indicated by the vertical solid line in **a** and **b**, respectively. The darker the grey, the higher the proportion is. Parameters are: 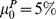, 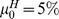, 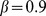, 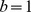 and 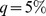.

The spatial spline correlograms estimated how the between-populations correlation was a function of spatial distance. Fig. 2 shows the correlograms for host genotype 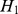 when 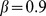. The profiles for the 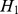 population dynamics exhibited a characteristic decrease of similarity (spatial autocorrelation) with distance with significantly positive autocorrelation at short spatial distances. Interestingly, when host and pathogen dispersed locally, the correlation dropped more quickly with distance than for intermediate dispersal distances (Fig. 2a, b). Thus, at intermediate mean dispersal distances, the metapopulation formed aggregates of local populations which displayed asynchronous dynamics - populations belonging to the same aggregate showed synchronised behaviour, and populations belonging to distinctly different aggregates followed different dynamics (Fig. 3). For large host and/or pathogen dispersal scales no spatial structure in correlation was observed (Fig. 2c) again indicating full synchrony of the metapopulation. Sasaki *et al.* [37] developed an island metapopulation model with migration occurring either globally or locally and found a similar pattern of population synchrony according to dispersal ability with a gene-for-gene epidemiological model. Thus, the increase in the size of population aggregates that behave similarly when dispersal increases is certainly not restricted to the case presented here. Note also the existence of negative correlations for intermediate distance, even for relatively high (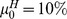) host dispersal.

### 3.2. Coexistence among pathogen genetic clusters

#### 3.2.1. Weak trade-off **(β = 0.9)**

**Figure 4:**
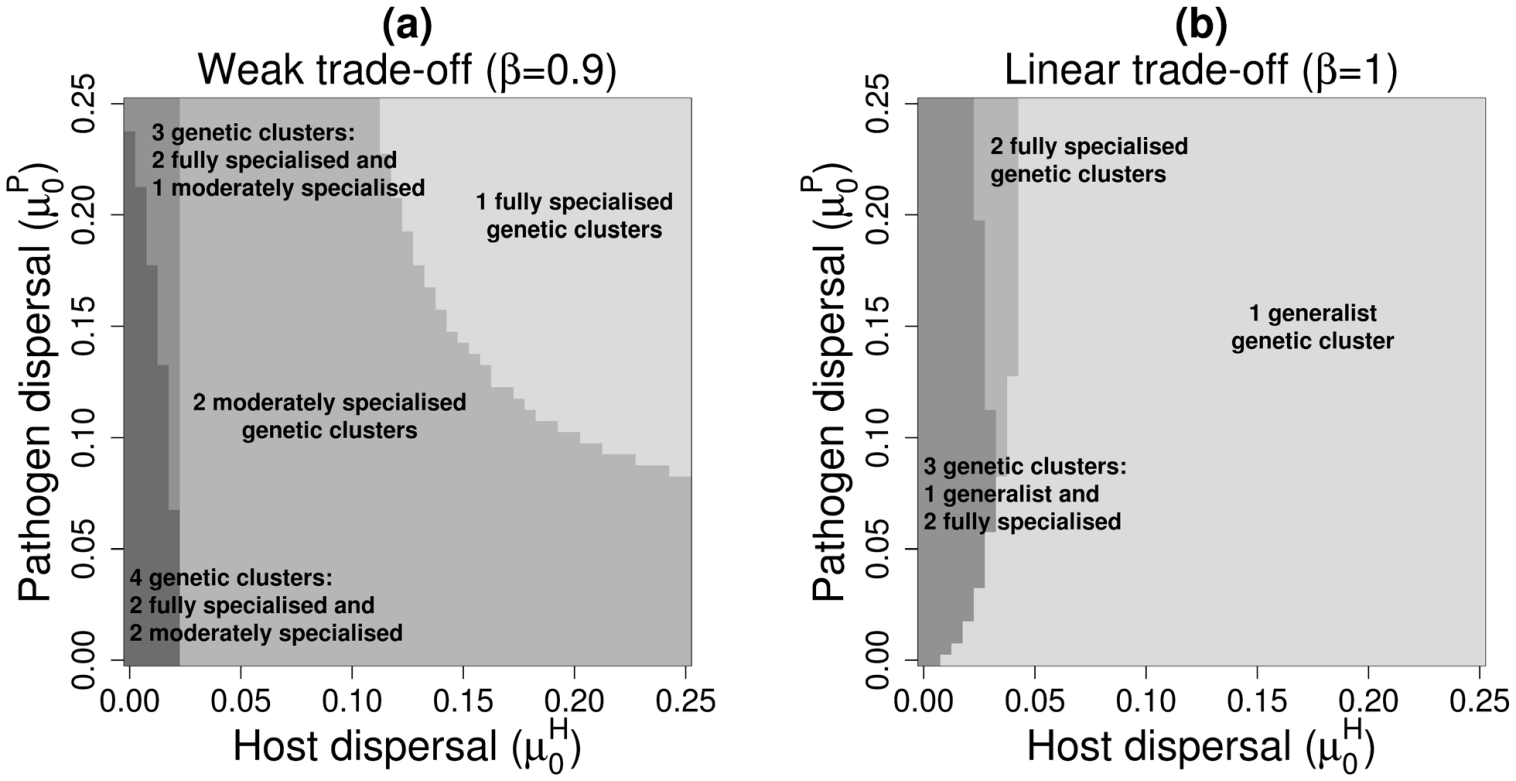
Stable coexistence among pathogen genetic clusters as a function of pathogen and host mean dispersal distances and for the case-study A. **a**: 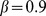; **b**: 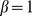. For clarity only the mean dispersal distances below 25% are displayed (see Fig. S4 in Text S1 for more details).

**Figure 5:**
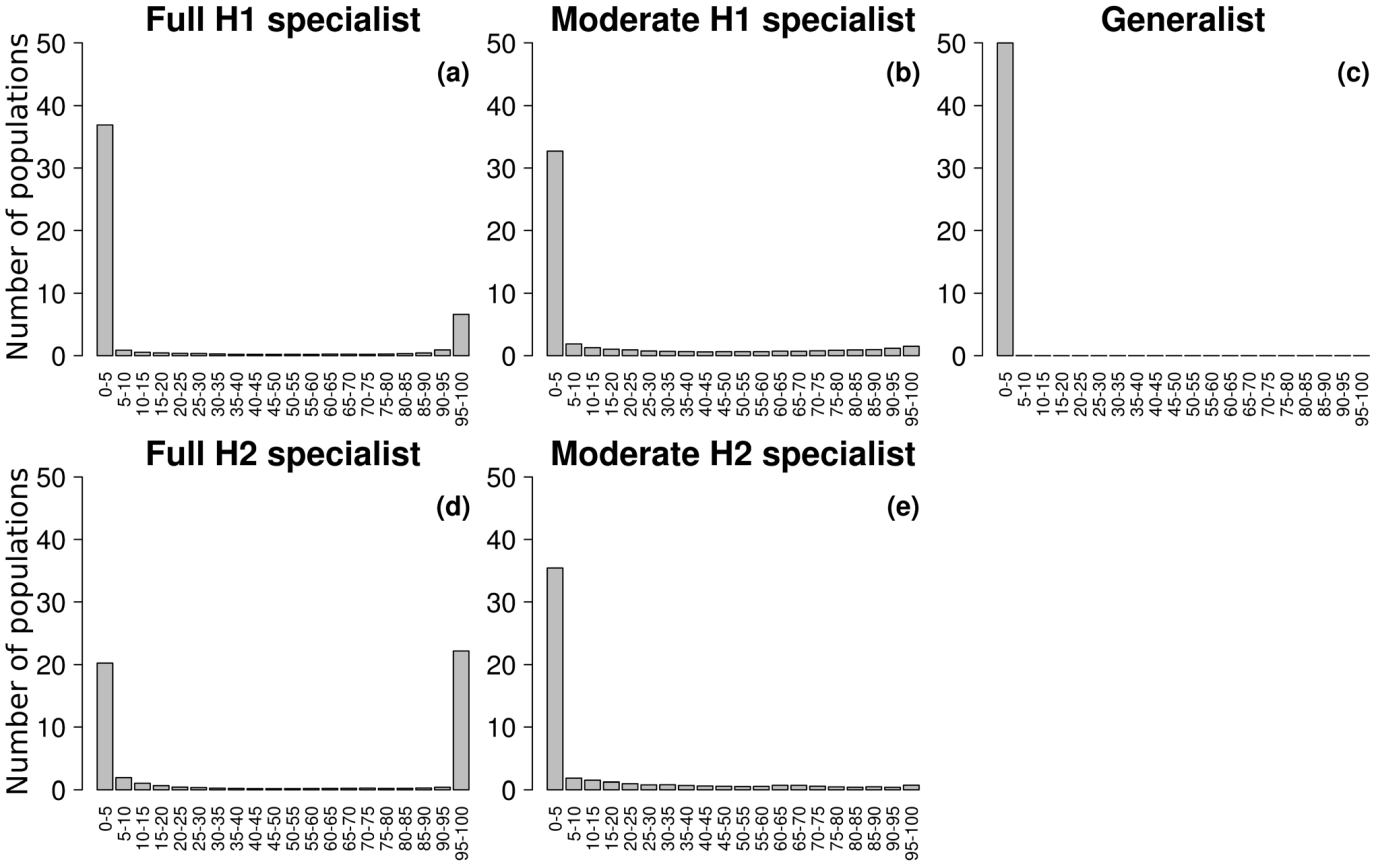
Distribution of the pathogen genetic clusters according to their proportion in each sub-population. This graph is issued from an example of simulation of the case-study A. The generalist cluster did not persist (**c**) and evolution led to the coexistence of the two fully (**a** and **d**) and two moderately (**b** and **e**) specialised clusters. Other parameters are: 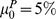, 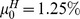 and 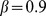 (see Fig. 1a for the global evolutionary trajectory).

When the gain of specialisation was greater than the cost of specialisation (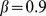, weak trade-off), up to four pathogen genetic clusters could coexist (Fig. 4a). For local host dispersal (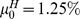) and up to intermediate pathogen mean dispersal distances (*i.e.* 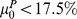), pathogen population diversity was maximal: the two fully specialised and two moderately specialised genetic clusters persisted (see also Fig. 1a). Other authors found the coexistence of more types than the number of habitats present in the environment. For example, Abrams [38] found that evolution can lead to the coexistence of one generalist and two specialist morphs when the system undergoes sustained fluctuations. Débarre and Lenormand [2] showed that restricted dispersal in heterogeneous environments favours stable coexistence among two highly and two moderately specialised morphs when the environment was composed of two habitats and due to habitat boundary polymorphism. In our case, when the host dispersed very locally, populations were largely asynchronous (Section 3.1). The pathogen metapopulation thus experienced a heterogeneous environment which made the simultaneous coexistence of a large number of genetic clusters possible, when pathogen dispersal was also limited (see Fig. S3 in Text S1 for an example). In addition, the repartition of each genetic cluster over the 50 populations indicated that the full specialists occupied some populations in very high proportion whereas the moderate specialists were more diffusely present and at lower proportion (Fig. 5). This was consistent with habitat boundary polymorphism [2]. Temporal fluctuations in the host frequencies probably reinforced this effect [38]. Finally, when host and pathogen dispersed very locally, local drift could lead to the extinction of one of the host genotypes in a few populations (see Fig. S3 in Text S1 for an example) potentially enhancing the maintenance of diversity in the host and pathogen metapopulation [39].

When pathogen dispersal ability increased (keeping a low 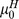 value), one of the moderately specialised genetic clusters went extinct resulting in a decrease in diversity. Indeed, increases in pathogen dispersal ability made the two fully specialised clusters more able to track changes in host frequencies across the metapopulation which enhanced their stability [40]. This implied a greater competition among the two moderately specialised genetic clusters leading to the extinction of one of them.

For large host and/or pathogen dispersal scales only one of the two fully specialised genetic clusters persisted. Under these dispersal conditions the metapopulation was totally synchronised and spatial coexistence was only transitory (Section 3.1). Thus, boom and bust dynamics (time periods characterised by sustained increase in one of the host genotype followed by its sharp and rapid contraction in favour of the second host genotype) dominated the system (see for an example Fig. 1c, d) leading to the extinction of one of the hosts (due to demographic stochasticity) and thus eliminating selection for the corresponding full specialist (Fig. 1c and d).

In all other dispersal contexts, evolution led to the selection of only two moderately specialised genetic clusters. Indeed, spatial heterogeneity experienced by the pathogen metapopulation decreased due to the formation of aggregates of local populations which displayed asynchronous dynamics (Section 3.1). Thus, spatial coexistence was also observed (Fig. 3c, d, e) but the total number of coexisting pathogen genetic clusters was smaller (Fig. 4). We give more insight to these cases in Section 3.3.1.

#### 3.2.2. Linear trade-off **(β = 1)**

The mechanisms explaining the pattern of pathogen diversity when 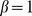 were the same as in the Section 3.2.1. We thus only describe here changes due to the trade-off shape.

When the trade-off shape was linear (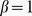), the two moderately specialised genetic clusters were replaced by a generalist genetic cluster (Fig. 1b *vs.* Fig. 1a). For local host dispersal (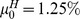 or 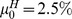) and up to intermediate pathogen mean dispersal distances (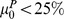) three pathogen genetic clusters could coexist: the generalist and the two fully specialised clusters (Fig. 4b and 1b). When pathogen mean dispersal distance increased (keeping a low 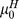 value), the generalist genetic cluster became unstable and only the two fully specialised genetic clusters were maintained. In all other dispersal contexts, the generalist genetic cluster alone persisted (see Section 3.3.2. for more details). For high host and pathogen dispersal abilities, the amplitude of oscillation between the host genotypes increased but to a lower extent than with a weak trade-off (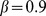) due to the higher attractiveness of more generalist genotypes. Thus, global extinctions of either host type were not observed with a linear trade-off, even if local extinctions occurred in small populations due to demographic stochasticity (not shown).

### 3.3. Specialisation level and variability of the genetic clusters

In this section we study the situations in which either the two moderately specialised (when 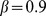) or the generalist (when 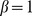) genetic clusters occurred (Fig. 4), and characterise their infection efficacy (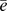) and homogeneity (efficacy range).

#### 3.3.1. Characterisation of the moderately specialised genetic clusters (β = 0.9)

**Figure 6:**
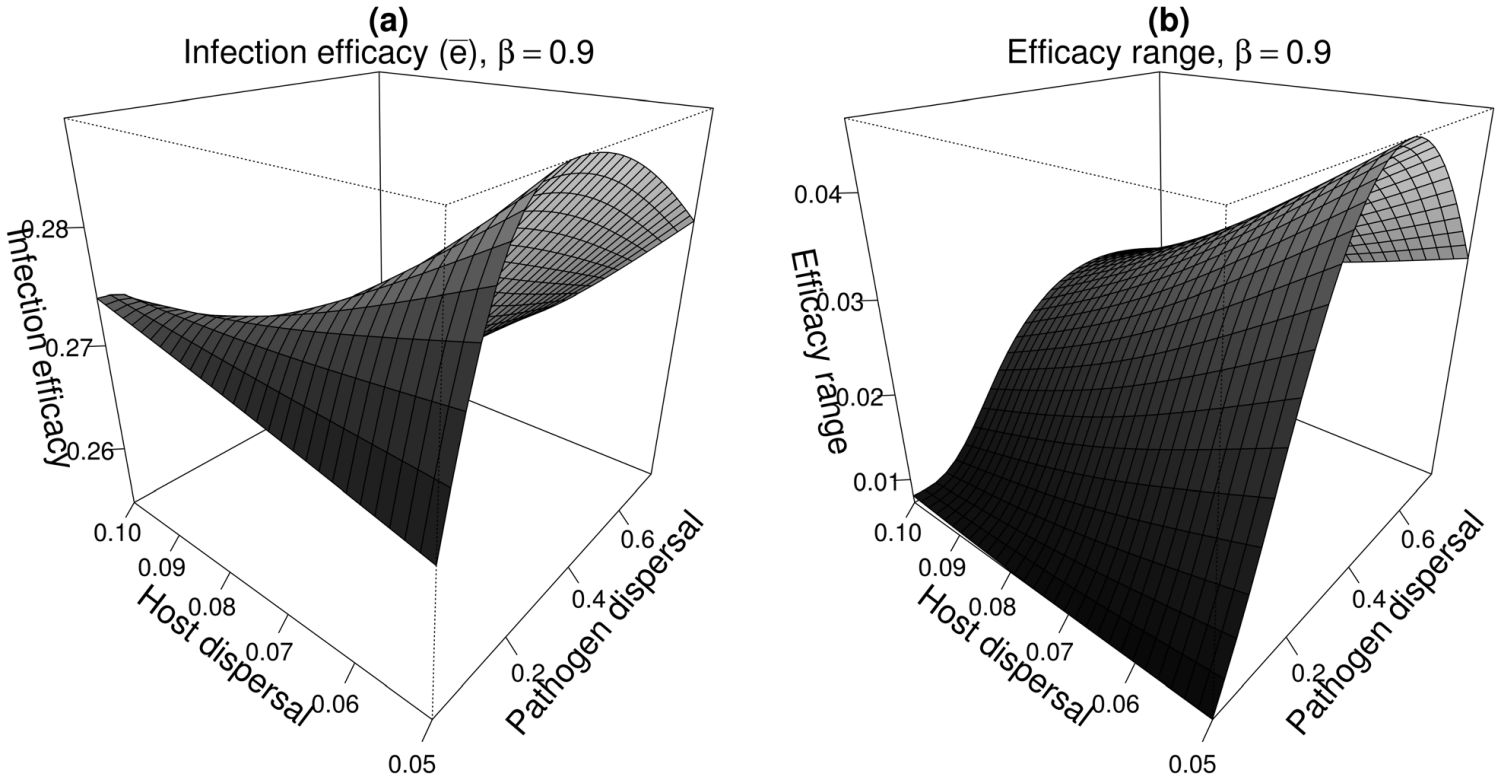
Infection efficacy 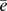 (a) and efficacy range (b) of the moderately specialised genetic clusters as a function of pathogen mean dispersal distance and for limited host dispersal (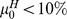). Parameters are those of the case-study A and 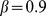. Only one genetic cluster is represented but two symmetric genetic clusters were present.

**Figure 7:**
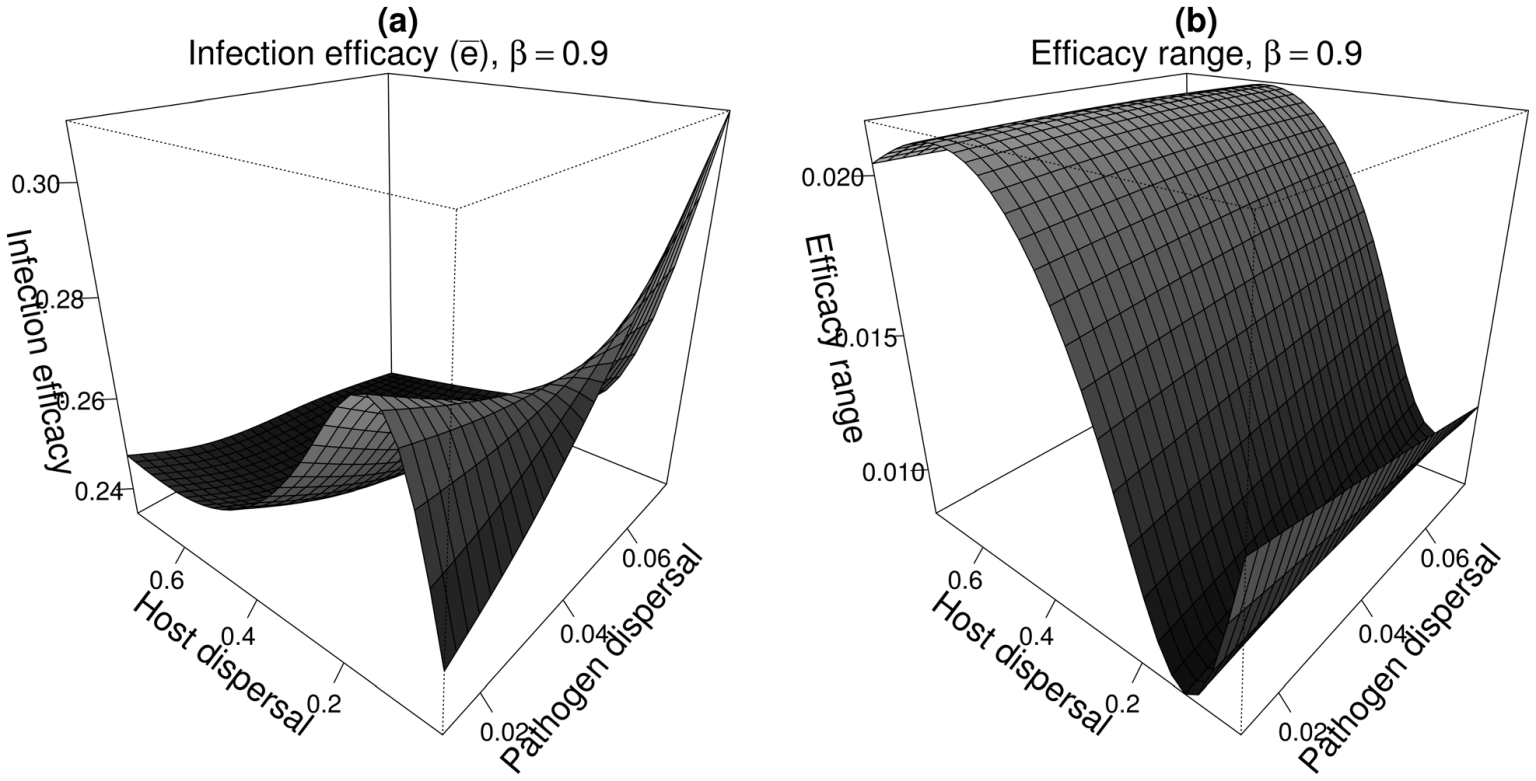
Infection efficacy 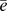 (a) and efficacy range (b) of the moderately specialised genetic clusters as a function of host mean dispersal distance and for limited pathogen dispersal (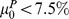). Parameters are those of the case-study A and 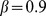. Only one genetic cluster is represented but two symmetric genetic clusters were present.

The characteristics of the moderately specialised genetic clusters were consistent with the global pattern of metapopulation synchrony. We used here the results of Section 3.1. (see also Figs. 2 and 3) as a guide line to interpret Figs. 6 and 7.

At low host and pathogen dispersal abilities, most of the populations are asynchronous. The pathogen metapopulation experienced thus a highly heterogeneous environment which favoured low 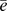 (Figs. 6 and 7, low 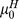 and 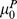 values).

Increases in the ability of the pathogen to disperse led generally to genetic clusters with the highest infection efficacy and efficacy range on their susceptible host (Fig. 6). Indeed, in that case the pathogen easily tracked spatial changes in host frequencies because of its higher dispersal ability.

**Figure 8:**
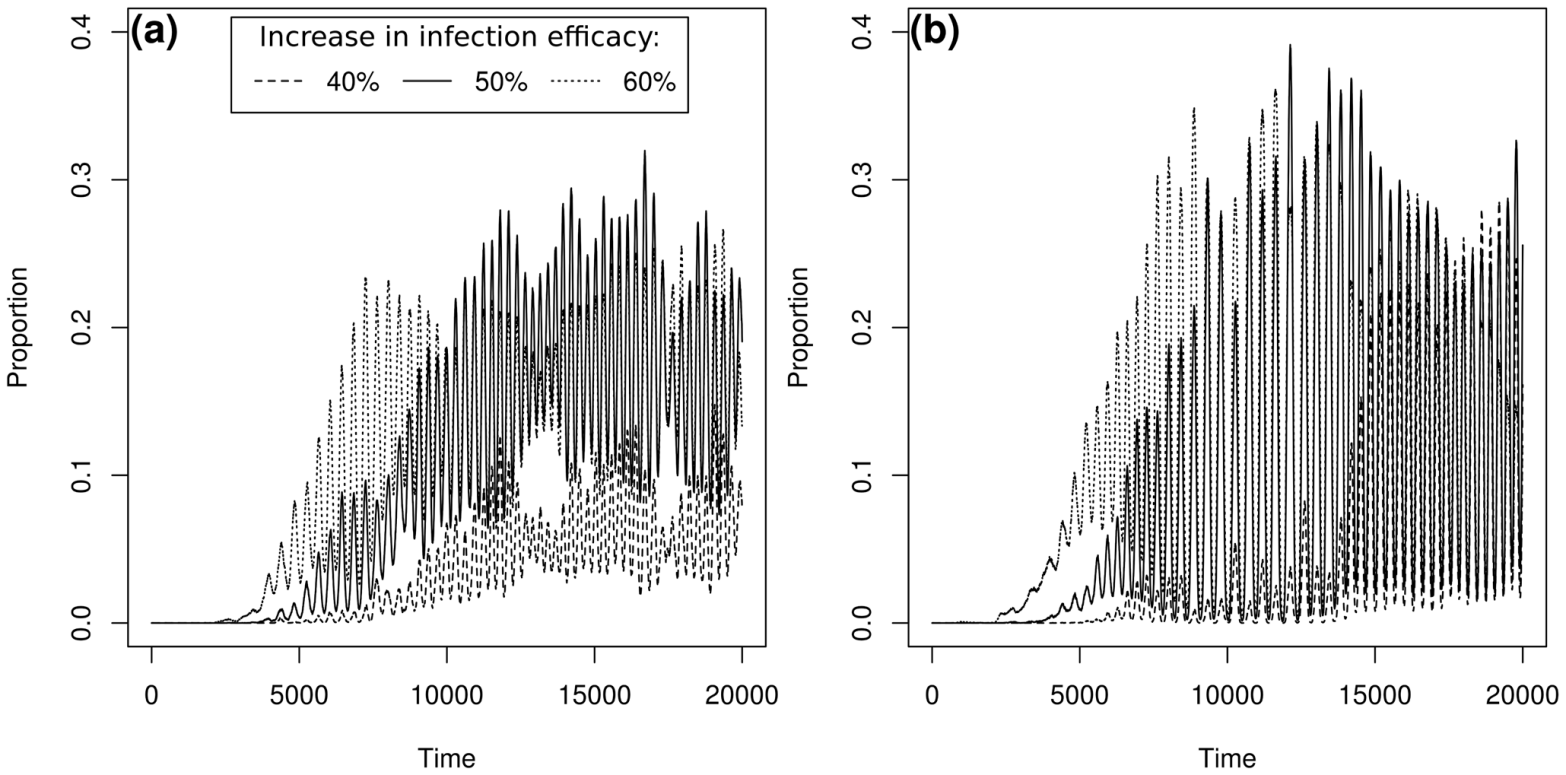
Examples of the dynamics of the main specialists present in the pathogen population. **a**: 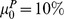; **b**: 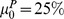. Parameters are those of case-study A, 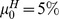 and 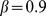.

Increases in the host dispersal ability first resulted in an increased aggregation among host populations with respect to both their dynamics and the distribution of host genotypes (Fig. 2). This resulted in an increase in the specialisation level of the pathogen genetic clusters (high infection efficacy and low efficacy range - Fig. 7) because the pathogen experienced a locally homogeneous environment (Fig. 3). For more global host dispersal the metapopulation as a whole became synchronised (Fig. 2) and the pathogen hardly tracked fluctuations in the frequencies of the two host genotypes (because 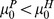). This resulted both in a decrease in 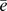 (Fig. 7a) and in an increase in efficacy range (Fig. 7b) due to the appearance of severe boom-and-bust dynamics in the host and pathogen metapopulation (Fig. 8).

#### 3.3.2. Characterisation of the generalist genetic cluster (β = 1)

**Figure 9:**
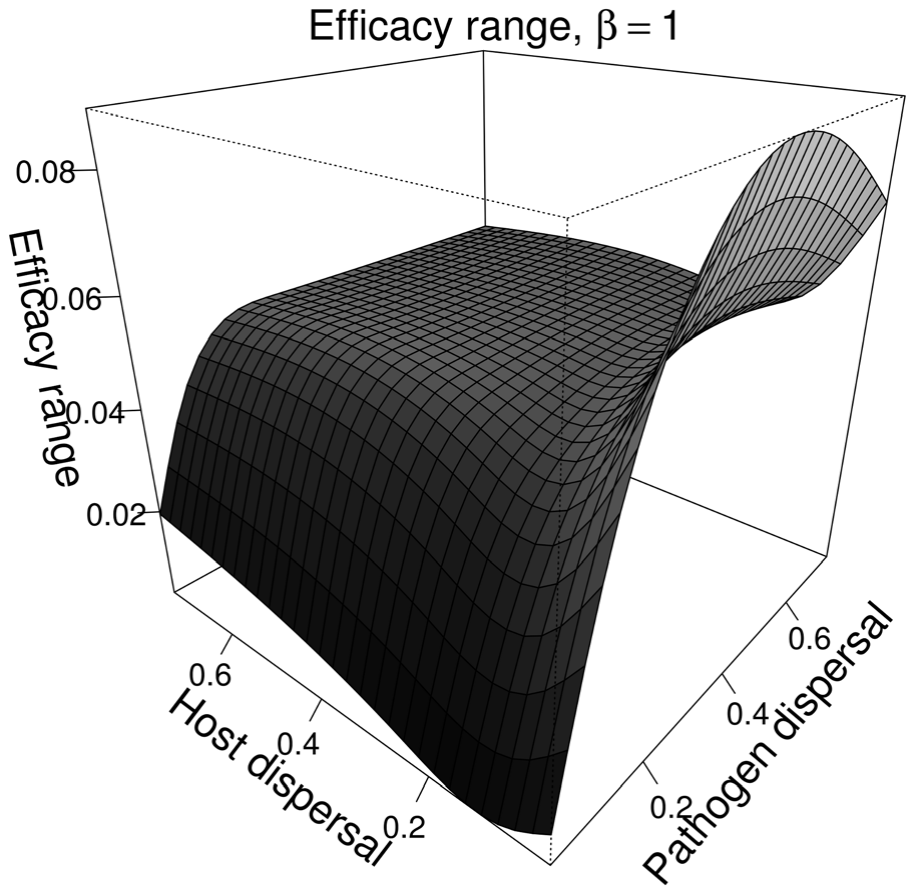
Efficacy range of the generalist genetic cluster as a function of pathogen and host mean dispersal distance. Parameters are those of the case-study A and 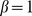.

The infection efficacy 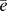 of the generalist genetic cluster did not vary with the mean dispersal distances of host and pathogen. Hence, we only present the effect of dispersal on the efficacy range of the generalist genetic cluster (Fig. 9).

**Figure 10:**
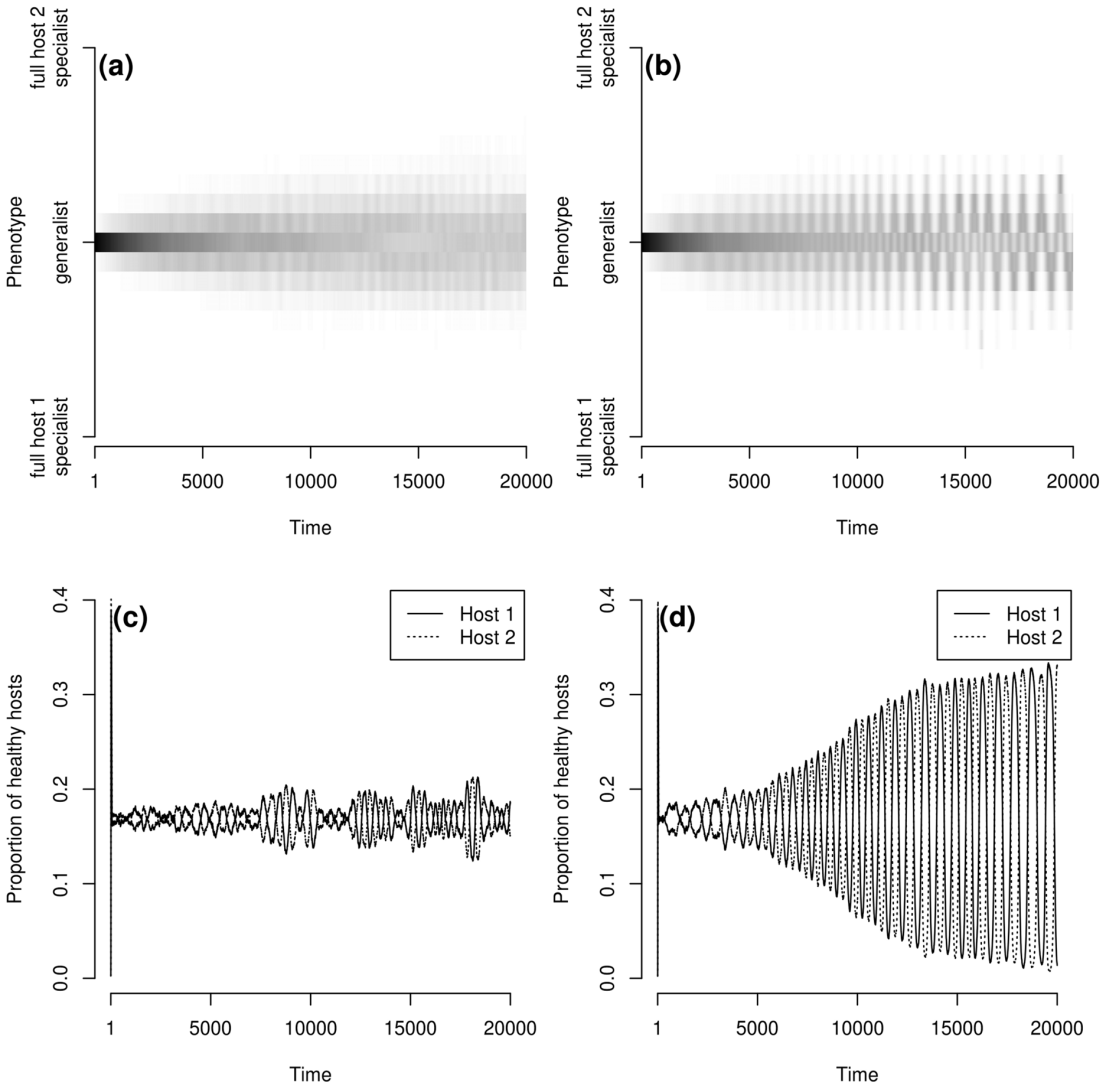
Examples of pathogen evolutionary trajectories (a and b) and the corresponding host dynamics (c and d, respectively). **a** and **c**: 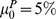; **b** and **d**: 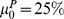. Parameters are those of case-study A, 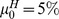 and 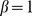.

When the pathogen mean dispersal distance increased, the efficacy range of the generalist genetic cluster increased up to a stable value which depended on the host mean dispersal distance (Fig. 9). As the pathogen dispersal ability increased the metapopulations acted as a single population where host and pathogen frequencies experienced growing oscillations [41]. Thus, the increase in efficacy range was due to the occurrence of severe reciprocal oscillations between more or less specialised pathogen genotypes (compare Fig. 10a and b) which accompanied similar oscillations between the two host genotypes (compare Fig. 10c and d).

In contrast, when host mean dispersal distance increased, we first observed a decrease in the efficacy range which was minimal when both host and pathogen had comparable dispersal scales (Fig. 9). Then, the efficacy range increased up to a plateau when host dispersal was greater than that of the pathogen. At large pathogen mean dispersal distances this increase did not occur and the efficacy range decreased before stabilising.

### 3.4. Sensitivity analysis

Overall, the 7 case-studies (Table 1) did not differ qualitatively and the patterns of pathogen diversity and evolution presented above were robust to changes in: the latency and infectious periods, pathogen reproduction, shape of the density dependence function, the 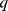 value, and the shape of the dispersal function.

#### 3.4.1. Coexistence among the genetic clusters

Increases in the latency and infectious period slightly reduced the diversity of the pathogen population for local host dispersal. However, coexistence among the two moderately specialised genetic clusters (weak trade-off) was enhanced because of a reduce amplitude in oscillations and thus a reduce probability of fixation of one of the two full specialists (Fig. S4 in Text S1). No clear effects of pathogen reproduction were found (Fig. S4 in Text S1). Using a linear instead of a sigmoid function of density dependence made coexistence easier by relaxing local competition for space (Fig. S5 in Text S1). Increases in 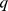 favoured less diversified pathogen populations. Conversely, a fat dispersal function favoured coexistence of several genotypes (Fig. S6 in Text S1). This could be explained by the proportion of autoinfection (infection of a local population by itself, Fig. S7 in Text S1) which was higher in the case of a fat dispersal function, and lower, in the case of higher 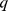 values. Indeed, a higher amount of autoinfection increased the level of asynchrony among local populations.

#### 3.4.2. Characteristics of the genetic clusters

When the trade-off was weak (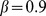, Figs. S8, S9, S10 and S11 in Text S1), increases in the latency period, in the 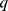 value and in the shape of the dispersal function decreased the specialisation level of the moderately specialised genetic clusters (decreased 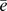). In contrast, due to the longer lifespan of an infectious plant, increases in the infectious period increased 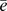. Changes in the others parameters did not change significantly the results respectively to case-study A. In addition, no clear differences were found on the heterogeneity of the genetic clusters. In the case where a generalist population was favoured (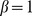, Figs. S12 and S13 in Text S1), the metapopulation was more heterogeneous and thus greater oscillations were observed for increased values of 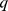 and pathogen reproduction. The other parameters have a negative effect on the efficacy range of the generalist pathogen genetic cluster.

## Discussion

This study assesses the demographic-genetic dynamics of a spatially explicit host-pathogen metapopulation in the absence of environmental heterogeneity. The host population was composed of two genotypes whereas the pathogen was able to evolve toward more or less specialised genotypes. We assessed the spatial structure of genotypic variation in pathogen diversity and the level of synchrony among populations. We then studied the influence of host and pathogen dispersal abilities on the evolution of pathogen specialisation and found the number of coexisting pathogen genetic clusters and their trait values under several strength of evolutionary trade-off and different dispersal scenarios of both organisms. Finally, we also assessed the sensitivity of these results to variations in pathogen life-history traits, in metapopulation structure and in dispersal function shape. Our key results are that restricted host dispersal can lead to a high level of pathogen diversity and that the degree of pathogen specialisation is strongly influenced by the spatial scale of host-pathogen interactions.

Pathogen metapopulation diversity evolved to its highest level when host and pathogen dispersal were both very local. Under those conditions, most populations were highly asynchronous with respect to disease dynamics leading to spatial coexistence among up to four pathogen genetic clusters. An increase in dispersal ability for both host and pathogen first resulted in the loss of the two fully specialised genetic clusters and selection for moderately specialised (or a generalist) genetic cluster(s) with low genotypic variability (efficacy range). Here also among-population asynchrony was sufficient to allow spatial coexistence. As the scale of dispersal increased, the system became increasingly dominated by boom-and-bust dynamics. Local populations became increasingly synchronised and spatial coexistence was only transitory. For large pathogen and host dispersal scales, severe oscillations appeared leading to frequent local extinction-recolonisation of hosts. When moderately specialised genetic clusters were selected, these oscillations also resulted in the global extinction of one of the hosts and the subsequent full specialisation of the pathogen population on the remaining host. In this case the metapopulation acted as a single population where host and pathogen frequencies experienced growing oscillations, resulting in a single genotype of each species being fixed.

The effect of host and pathogen dispersal on the maintenance of diversity was specifically studied by Thrall and Burdon [22] in a multi locus model. Their results confirmed that spatial structure is a crucial factor for explaining the levels of host and pathogen genetic diversity that are maintained in a metapopulation. In particular they found that restricted dispersal led to the highest diversity in terms of host resistance and pathogen infectivity genotypes. In addition, increases in the spatial scale of pathogen dispersal favoured more generalist pathogens carrying many infectivity genes. Those results are in line with the ones presented here showing that qualitative as well as quantitative interactions could lead to similar patterns of pathogen evolution. The conditions for the maintenance of genetic variation in a victim-exploiter system was also studied by Nuismer *et al.* [30], with a one-dimensional environment, and by Gavrilets and Michalakis [31], using an island model structure for the metapopulation. Both studies showed that when the environment was homogeneous, synchronisation among populations led to rapid loss of variation unless significant variation in allele frequencies was initially imposed. In addition, maintenance of variation required low migration rates, intermediate selection strength and the presence of a large number of populations. Asynchrony among populations and thus coexistence among genetic clusters was also found in our model to be favoured by low migration rates. However, the maintenance of polymorphism did not require that genotype frequencies varied among populations at initialisation, probably due to mutation, drift and the higher number of populations [31], [42]. For instance, we found that local drift can lead to the extinction of one of the host genotypes in a few populations potentially enhancing the maintenance of diversity in the host and pathogen metapopulation and contributing to asynchrony in dynamics [39].

The competitive exclusion principle suggests that in an environment composed of two resources, competition can either lead to selection for a generalist or for two specialists. We found here that up to four pathogen genetic clusters can coexist in a two-resource (host) environment. Using a one-patch model for studying adaptive evolution, Abrams [38] found that evolution can lead to such an output when the system undergoes sustained fluctuations (see also [43]). In addition, polymorphisms can be stabilised if direct frequency-dependent selection is negative, so that the net contribution of a given allele to fitness declines with increasing frequency [44]. Here we found that asynchrony among populations produces spatial heterogeneity in selection which implies negative direct frequency-dependent selection [42] and due to a restricted dispersal, favours stable coexistence among specialists and generalist morphs [2]. However, asynchrony in gene frequency fluctuation can develop even in the case of metapopulations where migration is occurring globally. In that case, a single population with asynchronous dynamics leads to a stable polymorphism at the metapopulation level [37]. This situation was not observed here.

A particular feature of the current study was to characterise the level of specialisation, as measured by 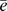, that evolves and persists in pathogen populations. When hosts disperse very locally, evolution favours the most specialised pathogen genotypes which can attack only one of the hosts. Indeed, local interactions due to local dispersal made easier the evolution of highly specialized pathogens as they adapted faster than less specialized pathogens (local growth rate was higher because of a higher infection efficacy) [45]. On the other hand, when host and/or pathogen dispersal scales are large, oscillations can lead to the extinction of one of the hosts resulting in the fixation of one of the full specialist pathogens. Otherwise, the specialisation level of the pathogen metapopulation depends on the spatial scale of host-pathogen interaction. When we fixed the host mean dispersal distance, the pathogen specialisation level was found to increase with pathogen dispersal capability, reaching its maximum when the pathogen dispersed more than the host.

The impact of gene flow on levels of pathogen specialisation has only rarely been discussed in the literature. Most studies deal with spatially unstructured populations [17], [46], [47], [48], assume qualitative interactions [16], [17] or focus on host life history traits [25], [26]. Best *et al.* [25] studied the conditions required for two specialised host genotypes to coexist but did not characterise the coexisting genotypes. Gandon [17] analysed a deterministic host-parasite coevolutionary model based on a modified matching allele model for genetic interactions. He found that the species with the higher migration rate evolved faster and was more likely to be locally adapted which is consistent with greater specialisation. Even if the properties of the two host genotypes do not change through time in our model, our results are consistent with Gandon's predictions since we found that the pathogen specialisation level was generally greater when it disperses more than the host.

A contrasting pattern of specialisation was observed when pathogen dispersal was fixed to be spatially local (mean dispersal distance ≤5% of the region size) and host dispersal scale was varied. Under these conditions, the pathogen generally dispersed less than the host and it was expected that this differential would decrease the level of pathogen specialisation. However, contrary to this prediction, increasing host mean dispersal distance initially resulted in greater pathogen specialisation, which reached its maximum for intermediate host mean dispersal distances (Fig. 7a). For larger host dispersal capabilities, the level of pathogen specialisation decreased. Recent results on the evolution of specialisation in spatially heterogeneous environments can explain this pattern of specialisation. Papaïx *et al.* [4] showed that the spatial distribution of habitats (hosts) impacts the mean phenotype of specialist morphs: specialist phenotypes are better adapted when habitat aggregation is high (see also [49]). In our case, for very local host dispersal, most local populations behaved asynchronously leading to a low aggregation of host genotypes in space which favoured low pathogen specialisation level. When host dispersal increased, we first observed increased aggregation among host populations with respect to both their dynamics and the distribution of host genotypes. This resulted in an increase in the specialisation level of the pathogen. For more global host dispersal the metapopulation as a whole became synchronised and the pathogen simply tracked fluctuations in the frequencies of the two host genotypes. In this last case, pathogen specialisation decreased because oscillations between host genotypes were too rapid to make it possible for the pathogen to fully adapt to its host – clearly this situation would favour generality.

In our study, although the host population was diversified, we assumed that the properties of the two host genotypes did not change through time and thus that the pathogen evolves faster than the host. This is particularly the case for crops, for which the same host genotypes are used for several years and that integrate relatively low genetic diversity for disease resistance. In agricultural landscapes host dispersal and aggregation is restricted to spatial and temporal patterns of cropping. Although there have been a few attempts to produce advice on optimal crop spatial organisation for restricting pathogen evolution (*e.g.* [50]; but see [51]), this question clearly still requires more investigation. Papaïx *et al.* [52] analysed the pathogenicity structure of leaf rust populations (*Puccinia triticina*) on wheat (*Triticum aestivum*) at the scale of France and found coexistence among qualitative specialists (very restricted host range), quantitative specialists (large host range but transmitted efficiently only by a few of them) and generalists (large host range with roughly equal preference among them). This high diversity of pathogenicity patterns is consistent with the high diversity found here when the host disperses essentially locally. In addition, we found, in the present study, that more generalist, and thus less damaging, pathogen genotypes were favoured when the host population fluctuates in time because, under these conditions, the pathogen population is forced to track host oscillations. Such fluctuations in variety frequencies could be impose in agricultural landscapes to prevent the evolution of pathogen populations toward highly specialised and adapted morphs. Moreover, local spatial aggregates of crops or varieties that behave similarly with regard to a given disease should be avoided in order to prevent the local emergence of specialised (and damaging) pathogen genotypes.

Another example is provided by invasive plants which are likely to exhibit low diversity with respect to disease resistance [53], [54]. In that context, the use of fungal pathogens as biocontrol agents has often been used against invasive weeds with several successful examples, but in a high number of cases bio-control has failed [55], [56]. Metapopulation processes and dispersal features are known to be important determinants in the success of biological control by parasites [57], [58]. By considering pathogen evolution, our results emphasise the importance of the spatial scale of interaction between host and pathogen as this may influence the level of pathogen adaptation to host genotypes and the nature of disease dynamics (whether or not boom-and-bust cycles occur), and thus the success of controlling a recently introduced host. However, the resistance structure of host populations can respond rapidly to selection pressures imposed by a pathogen [54] and further work must consider the coevolution of host and their parasite.

Relatively little is known about actual patterns of generalisation and specialisation in natural systems largely because many fewer studies have focused on the pathogenicity structure of pathogen populations [52], [59] than on the resistance structure of their host populations [12]. In general though, assessments of pathogen population structure have found multiple outcomes at the level of individual populations (monomorphic to highly polymorphic) – an observation likely reflecting a number of factors including local adaptation [22], fitness costs associated with pathogenicity [60] and stage in a frequency-dependent cycle of interaction between pathogen and host [61], as well as dispersal capability (isolation-by-distance) and life history features associated with effective local survival. The present approach gives insights into the role of host and pathogen dispersal in driving pathogen diversity and adaptation and encourages further characterisation of pathogenicity structure of crop and natural pathogen populations.

## Supporting Information

Text S1 Model description and supplementary figures.
(DOCX)
