# Supplementary material for: Evolution of Pathogen Specialisation in a Host Metapopulation: Joint Effects of Host and Pathogen Dispersal
Source: PLoS Comput Biol. 2014 May 22;10(5):e1003633. doi: 10.1371/journal.pcbi.1003633 (PMC4031062; doi:10.1371/journal.pcbi.1003633)
Supplement: Text S1 — Model description and supplementary figures. (DOCX) [file pcbi.1003633.s001.docx]

**Appendix S1: Model description**

We consider a metapopulation model in which plant and pathogen populations are inter-connected via dispersal of propagules (*e.g.* seeds and spores). The model describes a polycyclic disease caused by a foliar pathogen dispersed by air-borne spores (*e.g.* rust fungus). The model is stochastic and time is considered as discrete. In population $i$ and time $t$, individual plants are in one of the following states (SEI model): Susceptible ($S$), Exposed ($E$) and Infectious ($I$). In the following, model steps are described chronologically.

1. **Computation of newly produced spores and seeds:**

Infectious plants produce $r^{P}$ spores per day which belong to the same genotype as their parental lesion with probability $m_{pp}$. We assume that mutations from genotype $p$ to genotype $p'$ ($p\neq p'$) arise with probability $m_{pp'}$. In population $i$ and time $t$ the row $p$ of the matrix $M_{i}\left(t\right)$ giving the number of spores produced by the pathogen genotype $p$ writes:

$\left[M_{i}\left(t\right)\right]_{p.}\text{\textasciitilde Multi}\left(r^{P}\sum_{h} I_{ihp}\left(t-1\right),\left[m_{p1},m_{p2},\cdots\right]\right)$.

Thus, the total number of spores, $Sp_{ip}\left(t\right)$, belonging to the pathogen genotype $p$ and produced in population $i$ and time $t$ is computed as:

.

The host population is composed of two resistant genotypes, $H_{1}$ and $H_{2}$, with no mutations between them. In population $i$, susceptible plants produce $r^{H}$ seeds (new susceptible plants) per day giving thus:

$Se_{ih}\left(t\right)=r^{H}S_{ih}\left(t-1\right)$, for $h=H_{1},H_{2}$.

Where $Se_{ih}\left(t\right)$ is the number of seeds produced by the host $h$ in population $i$ and time $t$.

1. **Seeds and spores dispersal:**

Spores and seeds migrate from population $i$ to population $j$ with probability $\mu_{ij}^{P}$ and $\mu_{ij}^{H}$, respectively. At time $t$, the row $i$ of the dispersal matrix $D_{p}^{P}\left(t\right)$ (respectively, $D_{h}^{H}\left(t\right)$) giving the number of spores of type $p$ (respectively, seeds of type $h$) dispersing from patch $i$ to the others populations writes:

$\left[D_{p}^{P}\left(t\right)\right]_{i.}\text{\textasciitilde Multi}\left(Sp_{ip}\left(t\right),\left[\mu_{i1}^{P},\mu_{i2}^{P},\cdots\right]\right)$ and $\left[D_{h}^{H}\left(t\right)\right]_{i.}\text{\textasciitilde Multi}\left(Se_{ih}\left(t\right),\left[\mu_{i1}^{H},\mu_{i2}^{H},\cdots\right]\right)$.

Thus, the total numbers of spores ($Sp_{ip}^{tot}\left(t\right)$) and seeds ($Se_{ih}^{tot}\left(t\right)$) arriving in population $i$ at time $t$ are computed as:

$Sp_{ip}^{tot}\left(t\right)=\sum_{j} \left[D_{p}^{P}\left(t\right)\right]_{ji}$ and $Se_{ih}^{tot}\left(t\right)=\sum_{j} \left[D_{h}^{H}\left(t\right)\right]_{ji}$.

1. **Contamination of susceptible plants:**

Spores arriving on a host population contaminate a susceptible plant with probability $\pi_{i}^{P}\left(x\right)$, where $\pi_{i}^{P}\left(\cdot\right)$ is an increasing function of $x=\sum_{h} S_{ih}\left(t-1\right)/\sum_{h} \left(S_{ih}\left(t-1\right)+E_{ih}\left(t-1\right)+I_{ih}\left(t-1\right)\right)$, the proportion of susceptible plants in the population at time $t-1$. The number of new possible infections (spores that enter in contact with a susceptible plant), $NewCont_{i}\left(t\right)$, in population $i$ and time $t$ is first computed regardless the host and pathogen genotypes as:

$NewCont_{i}\left(t\right)\sim\text{Bin}\left(\sum_{h} S_{ih}\left(t-1\right),\pi_{i}^{P}\left(x\right)\right)$.

Then, the new possible infections are dispatched among the pathogen genotypes according to their proportion in the set of spores arriving in the population $i$ and time $t$, , and following a multinomial distribution:

.

The number of new possible infections, $\left[NewContP_{i1}\left(t\right),NewContP_{i2}\left(t\right),\cdots\right]$, is then updated by taking the minimum between $\left[NewContP_{i1}\left(t\right),NewContP_{i2}\left(t\right),\cdots\right]$ and the actual number of spores arriving in that population to insure that the number of possible infections is lower than the number of spores arriving on the population:

$\left[NewContP_{i1}\left(t\right),NewContP_{i2}\left(t\right),\cdots\right]=\text{Min}\left(\left[NewContP_{i1}\left(t\right),NewContP_{i2}\left(t\right),\cdots\right],\left[Sp_{i1}^{tot}\left(t\right),Sp_{i2}^{tot}\left(t\right),\cdots\right]\right)$.

The new possible infections are then dispatched among the host genotypes according to their proportion in the susceptible plant population, , and following a multinomial distribution leading for the pathogen genotype $p$ to:

$\left[NewContPH_{ipH_{1}}\left(t\right),NewContPH_{ipH_{2}}\left(t\right)\right]\sim\text{Multi}\left(NewContP_{ip}\left(t\right),\left[\frac{S_{iH_{1}}\left(t-1\right)}{S_{iH_{1}}\left(t-1\right)+S_{iH_{2}}\left(t-1\right)},\frac{S_{iH_{2}}\left(t-1\right)}{S_{iH_{1}}\left(t-1\right)+S_{iH_{2}}\left(t-1\right)}\right]\right)$.

Again, the minimum between $\left[NewContPH_{ipH_{1}}\left(t\right),NewContPH_{ipH_{2}}\left(t\right)\right]$ and the actual number of susceptible plants in the population is taken to insure that the number of possible infections is lower than the number of susceptible plants in the population:

$\left[NewContPH_{ipH_{1}}\left(t\right),NewContPH_{ipH_{2}}\left(t\right)\right]=\text{Min}\left(\left[NewContPH_{ipH_{1}}\left(t\right),NewContPH_{ipH_{2}}\left(t\right)\right],\left[S_{iH_{1}}\left(t-1\right),S_{iH_{2}}\left(t-1\right)\right]\right)$.

1. **Infection of susceptible plants (transition from susceptible (S) to exposed (E) plants):**

A susceptible plant receiving a spore (contaminated plant) becomes infected with a probability $e_{ph}$, the infection efficacy of pathogen genotype $p$ on host genotype $h$. We thus have:

.

1. **Transition from exposed (E) to infectious (I) plants:**

Once infected, the plant remains latent during $\tau$ days before becoming infectious. The transition from exposed to infectious plants is given by:

$\left\{\begin{matrix} NewIplants_{iph}\left(t\right)\text{\textasciitilde Bin}\left(E_{iph}\left(t-1\right),1-\exp\left(-\frac{1}{\tau}\right)\right) \\ I_{iph}\left(t\right)=I_{iph}\left(t-1\right)+NewIplants_{iph}\left(t\right) \\ E_{iph}\left(t\right)=E_{iph}\left(t\right)-NewIplants_{iph}\left(t\right) \end{matrix}\right.$.

1. **Removal of infectious hosts:**

After T days of sporulation (infectious period), the plant is removed. The transition from infectious plants to removed plants is given by:

$\left\{\begin{matrix} NewRplants_{iph}\left(t\right)\text{\textasciitilde Bin}\left(I_{iph}\left(t-1\right),1-\exp\left(-\frac{1}{T}\right)\right) \\ I_{iph}\left(t\right)=I_{iph}\left(t\right)-NewRplants_{iph}\left(t\right) \end{matrix}\right.$.

1. **Removal of susceptible hosts:**

In the population $i$ and time $t$ and for the host genotype $h$, susceptible plants that die, $DeadPlants_{ih}\left(t\right)$, are removed according to a binomial distribution with parameters $S_{ih}\left(t-1\right)$ and $d^{H}$ leading to:

$\left\{\begin{matrix} DeadPlants_{ih}\left(t\right)\text{\textasciitilde Bin}\left(S_{ih}\left(t\right),d^{H}\right) \\ S_{ih}\left(t\right)=S_{ih}\left(t\right)-DeadPlants_{ih}\left(t\right) \end{matrix}\right.$.

1. **Establishment of new susceptible plants:**

Seeds get established in population $i$ and time $t$ with probability $\pi_{i}^{H}=1-\sum_{h} \left(S_{ih}\left(t\right)+E_{ih}\left(t\right)+I_{ih}\left(t\right)\right)/K_{i}$, where $K_{i}$ is the carrying capacity of population $i$. The number of new susceptible plants, $NewPlants_{i}\left(t\right)$, that get successfully established in population $i$ and time $t$ is first computed regardless the host genotype as:

$NewPlants_{i}\left(t\right)\sim\text{Bin}\left(K_{i}-\sum_{h} \left(S_{ih}\left(t\right)+E_{ih}\left(t\right)+I_{ih}\left(t\right)\right),\pi_{i}^{H}\right)$.

Then, the new individual plants are dispatched among the host genotypes according to their proportion in the set of seeds arriving in the population $i$ and time $t$, , and following a multinomial distribution:

$\left[NewPlants_{iH_{1}}^{*}\left(t\right),NewPlants_{iH_{2}}^{*}\left(t\right)\right]\sim\text{Multi}\left(NewPlants_{i}\left(t\right),\left[\frac{Se_{iH_{1}}^{tot}\left(t\right)}{Se_{iH_{1}}^{tot}\left(t\right)+Se_{iH_{2}}^{tot}\left(t\right)},\frac{Se_{iH_{2}}^{tot}\left(t\right)}{Se_{iH_{1}}^{tot}\left(t\right)+Se_{iH_{2}}^{tot}\left(t\right)}\right]\right)$.

The number of susceptible sites in population $i$ and time $t$ is then updated by taking the minimum between $\left[NewPlants_{iH_{1}}^{*}\left(t\right),NewPlants_{iH_{2}}^{*}\left(t\right)\right]$ and the actual number of seeds arriving in that population to insure that the number of newly established plants is lower than the number of seeds arriving on the population:

$\left\{\begin{matrix} S_{iH_{1}}\left(t\right)=S_{iH_{1}}\left(t\right)+\text{Min}\left(NewPlants_{iH_{1}}^{*}\left(t\right),Se_{iH_{1}}^{tot}\left(t\right)\right) \\ S_{iH_{2}}\left(t\right)=S_{iH_{2}}\left(t\right)+\text{Min}\left(NewPlants_{iH_{2}}^{*}\left(t\right),Se_{iH_{2}}^{tot}\left(t\right)\right) \end{matrix}\right.$.


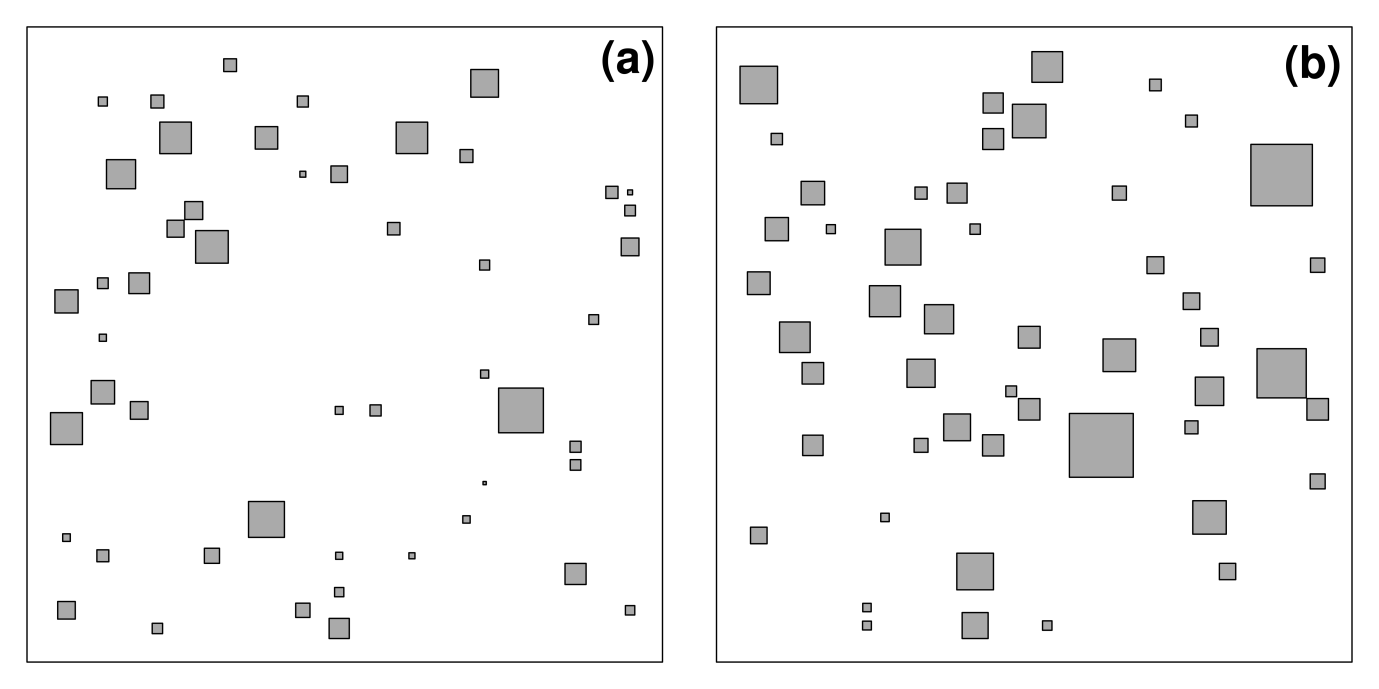


**Figure S1** Examples of metapopulation structures with 50 populations that cover $q=5\%$ (**a**) or $q=10\%$ (**b**) on the environment.


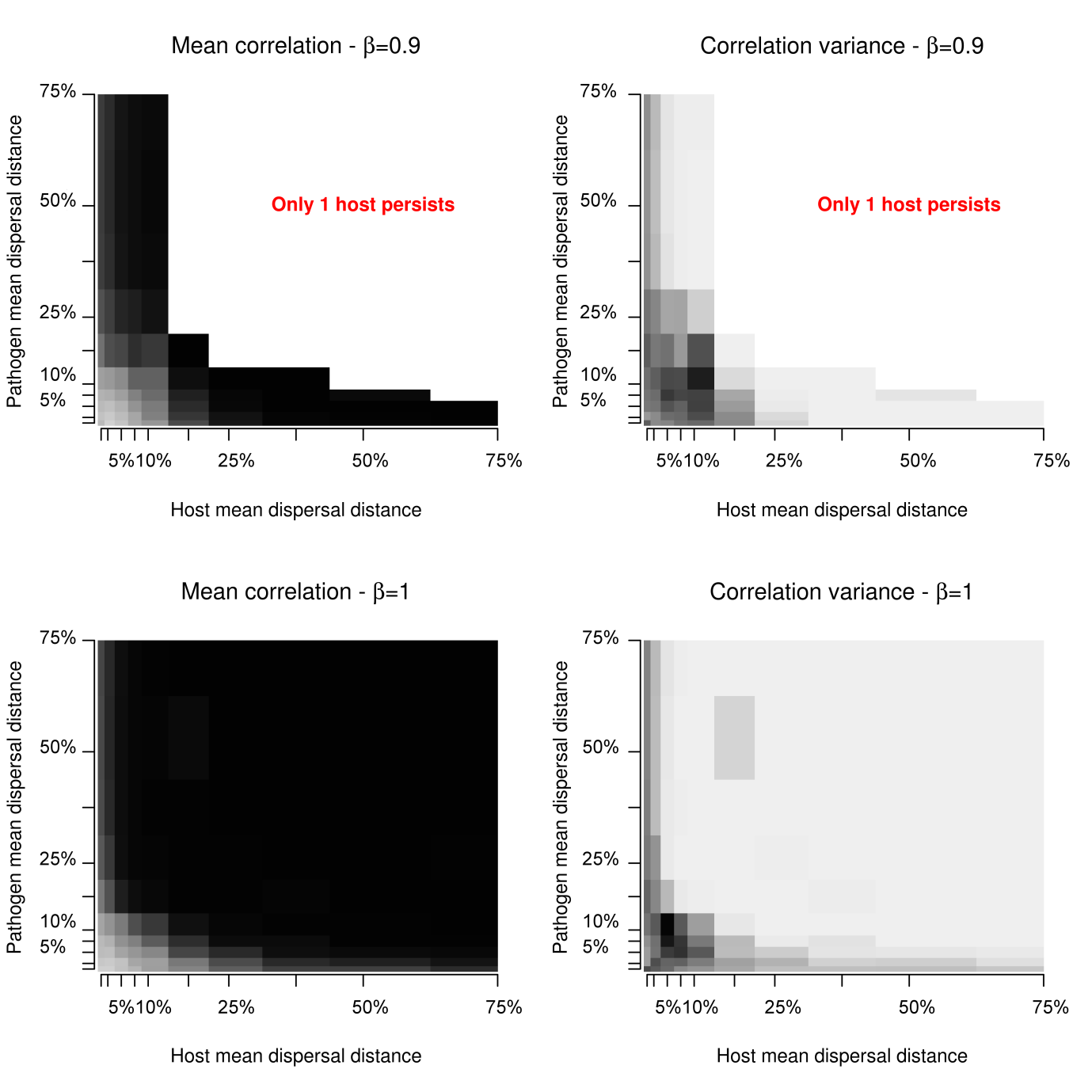


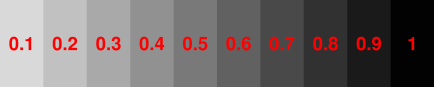

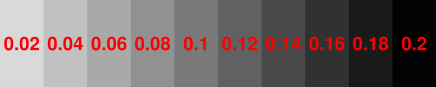


**Figure S2** Mean correlation and correlation variance among the global host metapopulation dynamics and the local host dynamics based on the 20 replicates. The higher the mean correlation, the more synchronized the metapopulation.


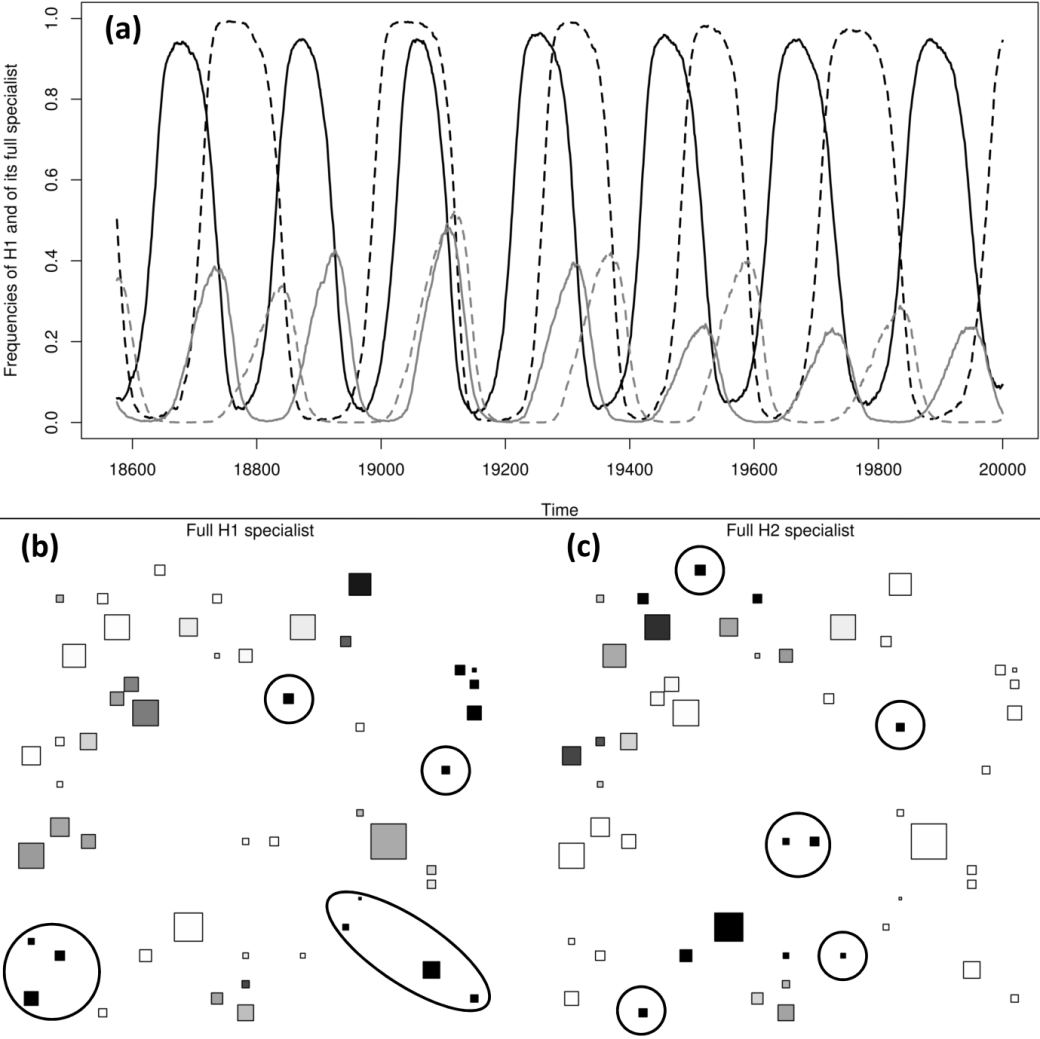


**Figure S3** Example of population asynchrony. The top row displays the dynamics of $H_{1}$ and of its full pathogen specialist on two different populations. The bottom row displays the population spatial structure. **a**: black, $H_{1}$ frequency; light grey, $H_{1}$ specialist frequency. **b** and **c**: full $H_{1}$ specialist and full $H_{2}$ specialist proportions at the population level, respectively. The darker the grey, the higher the proportion is. Black circles indicate the populations were one host goes extinct. Parameters are: $\mu_{0}^{P}=5\%$, $\mu_{0}^{H}=1.25\%$, $\beta=0.9$, $b=1$ and $q=5\%$.


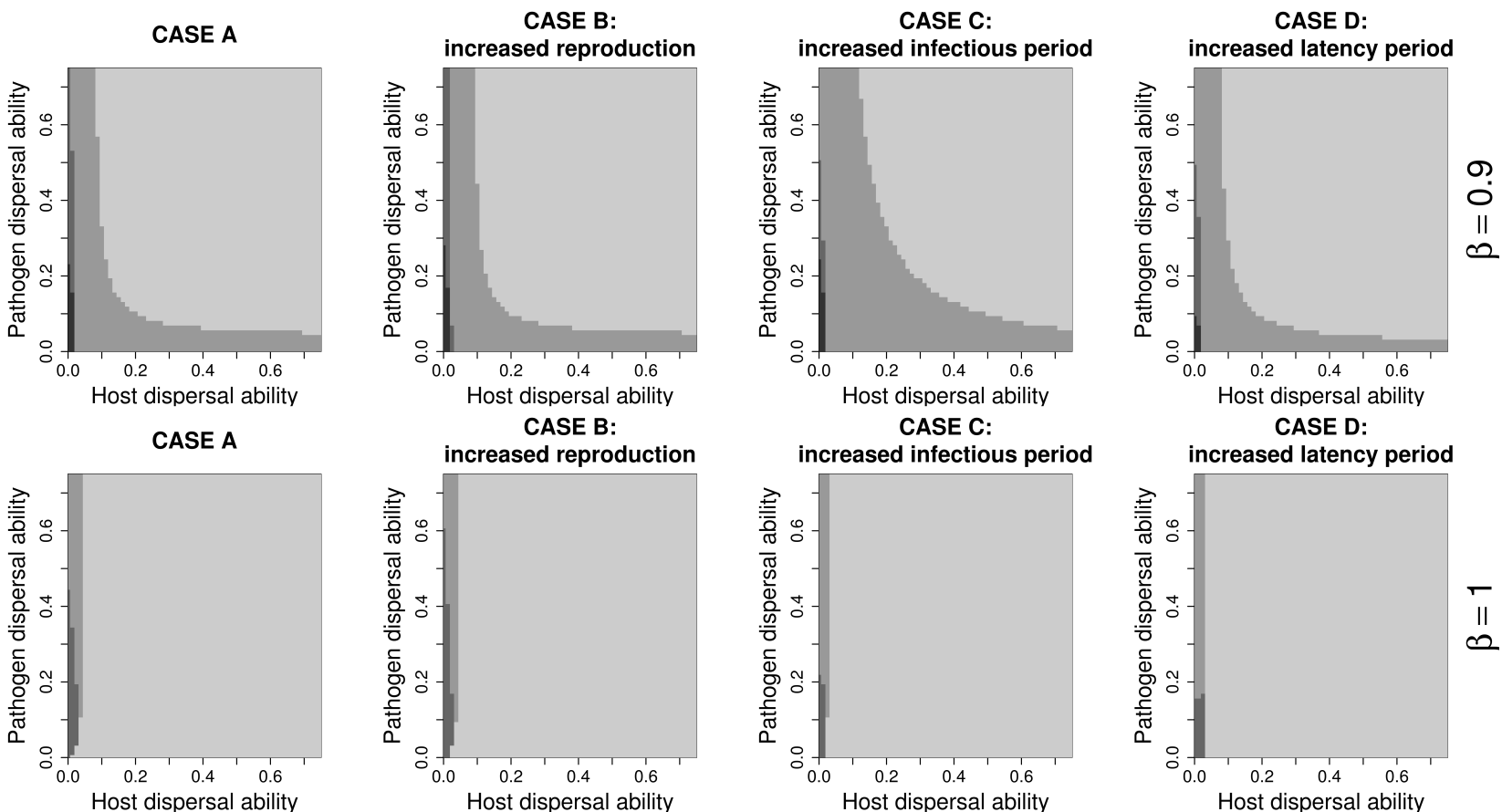


**Figure S4** Stable coexistence among pathogen phenotypes as a function of pathogen and host mean dispersal distances and for the case-studies A to D.

**
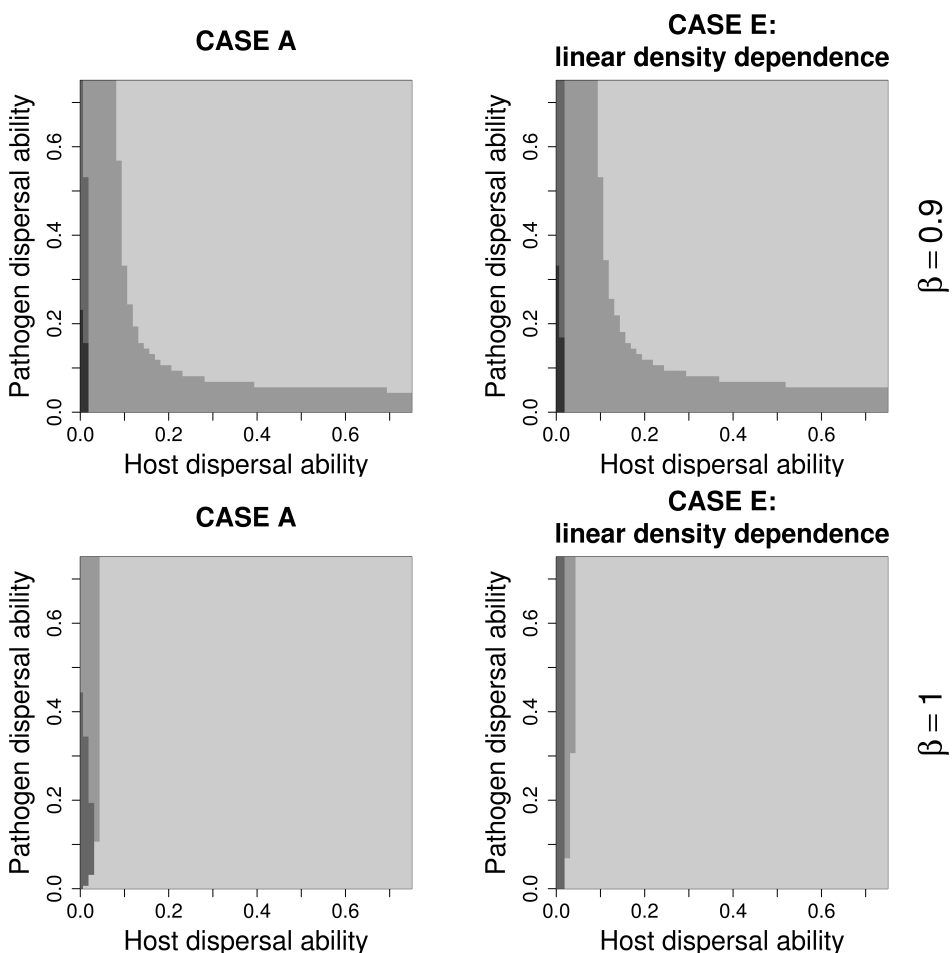
**

**Figure S5** Stable coexistence among pathogen phenotypes as a function of pathogen and host mean dispersal distances and for the case-studies A and E.


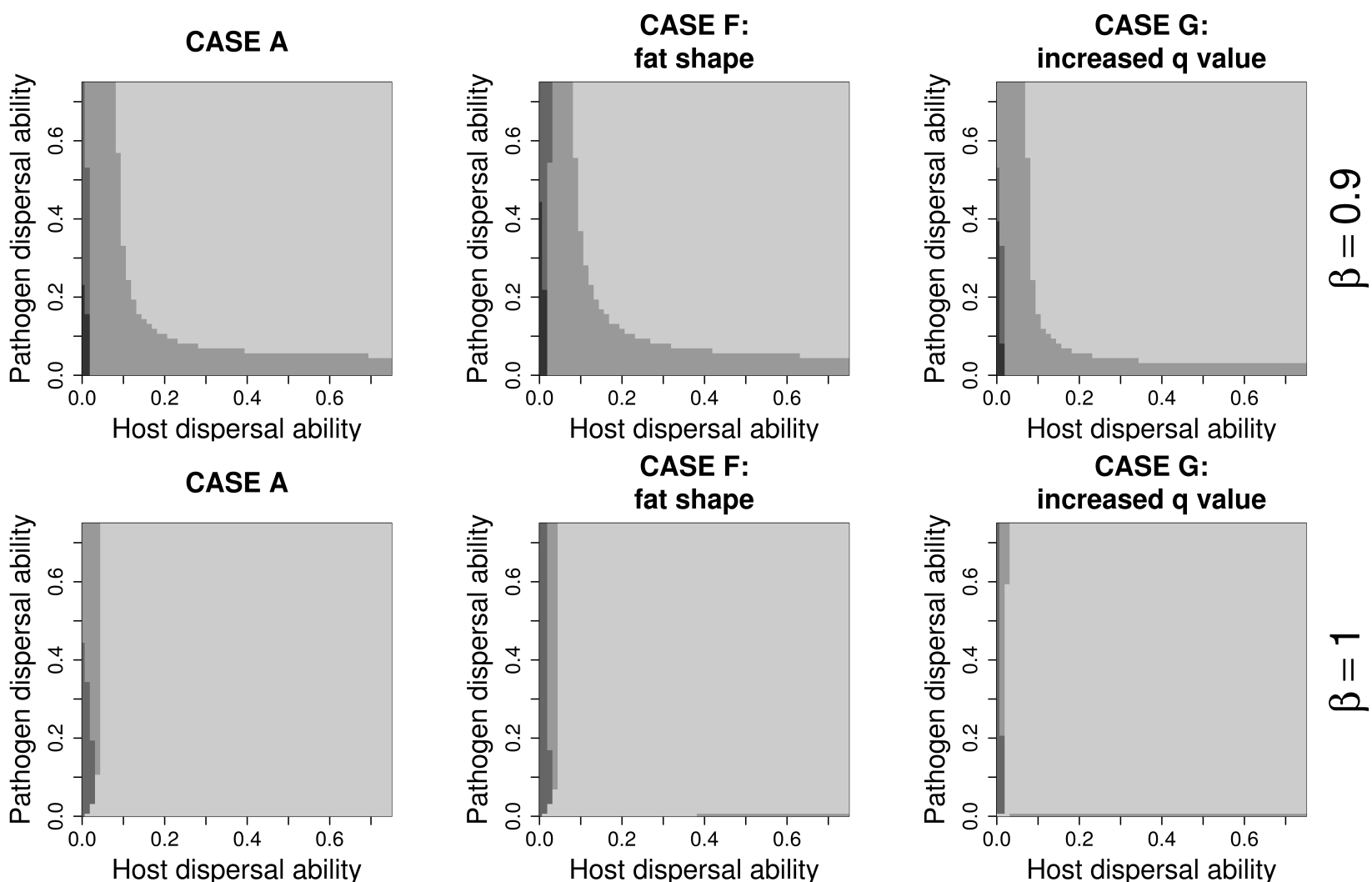


**Figure S6** Stable coexistence among pathogen phenotypes as a function of pathogen and host mean dispersal distances and for the case-studies A, F and G.


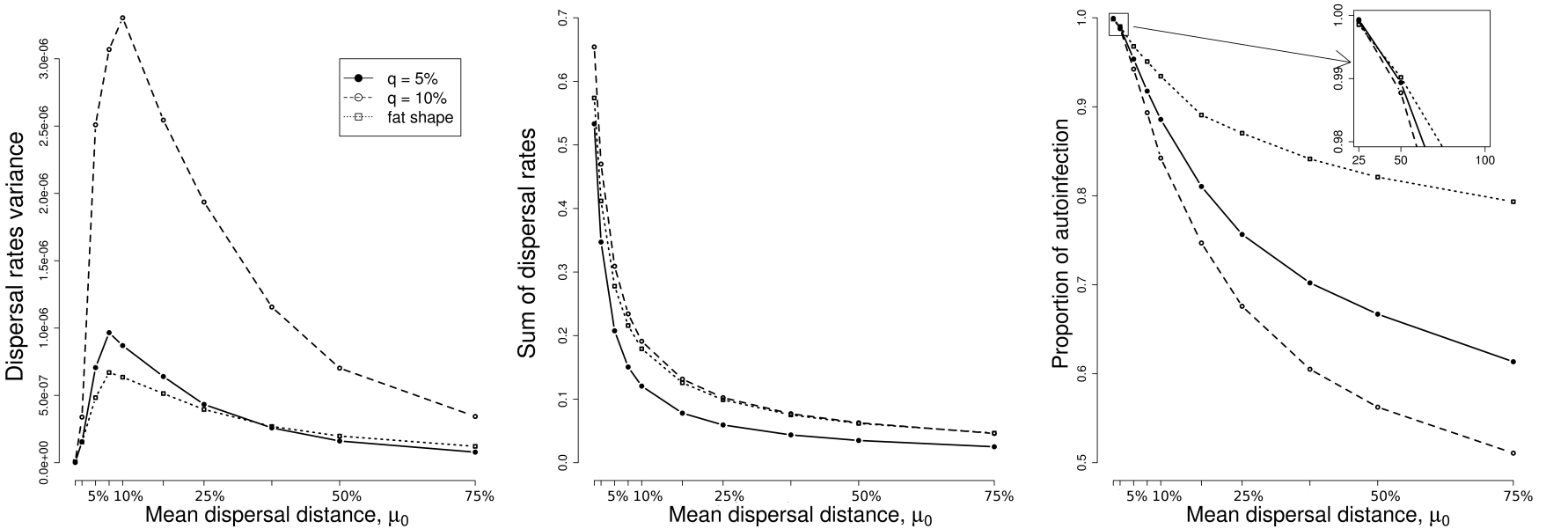


**Figure S7** From left to right: dispersal rates variance, sum of dispersal rates ($\text{mean}\left( \sum_{j=1}^{50} \mu_{ij} \right)$) and proportion of autoinfection ($\text{mean}\left( \frac{\mu_{ii}}{\sum_{j=1}^{50} \mu_{ji}} \right)$) as a function of the mean dispersal distance.


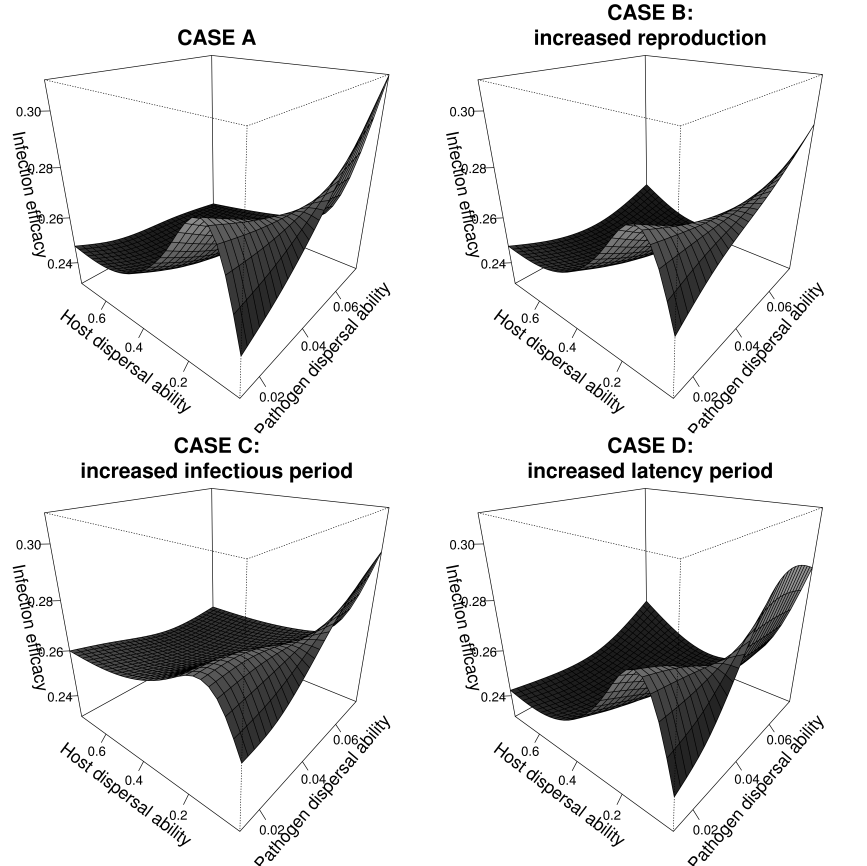


**Figure S8** Infection efficacy of the pathogen genetic clusters ($\bar{e}$) when 2 genetic clusters coexisted ($\beta=0.9$).


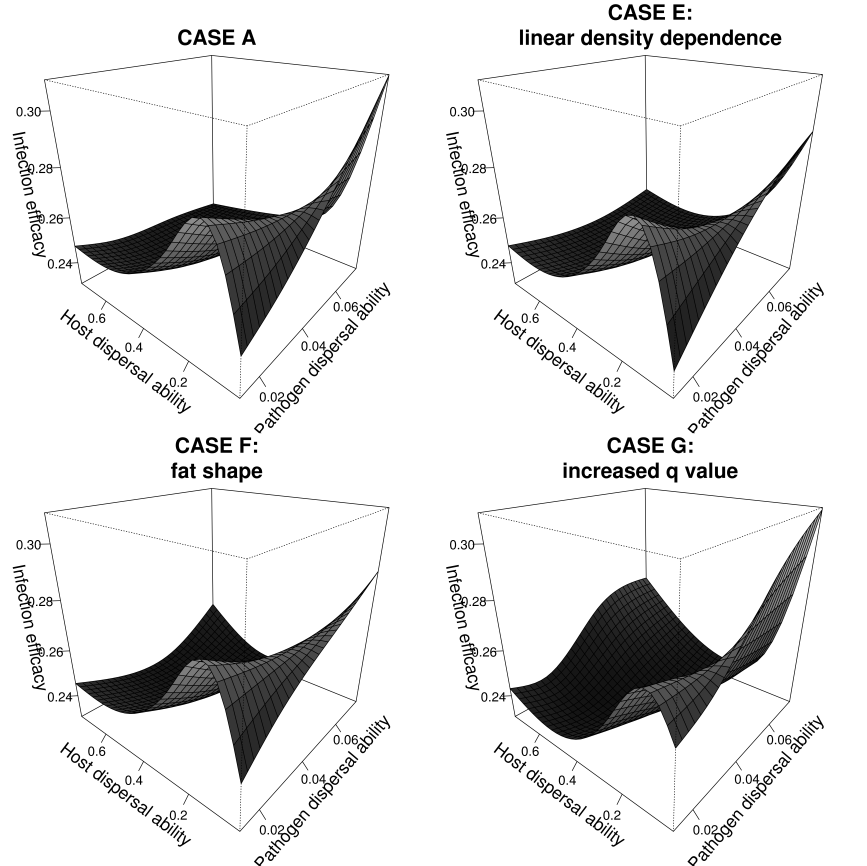


**Figure S9** Infection efficacy of the pathogen genetic clusters ($\bar{e}$) when 2 genetic clusters coexisted ($\beta=0.9$).


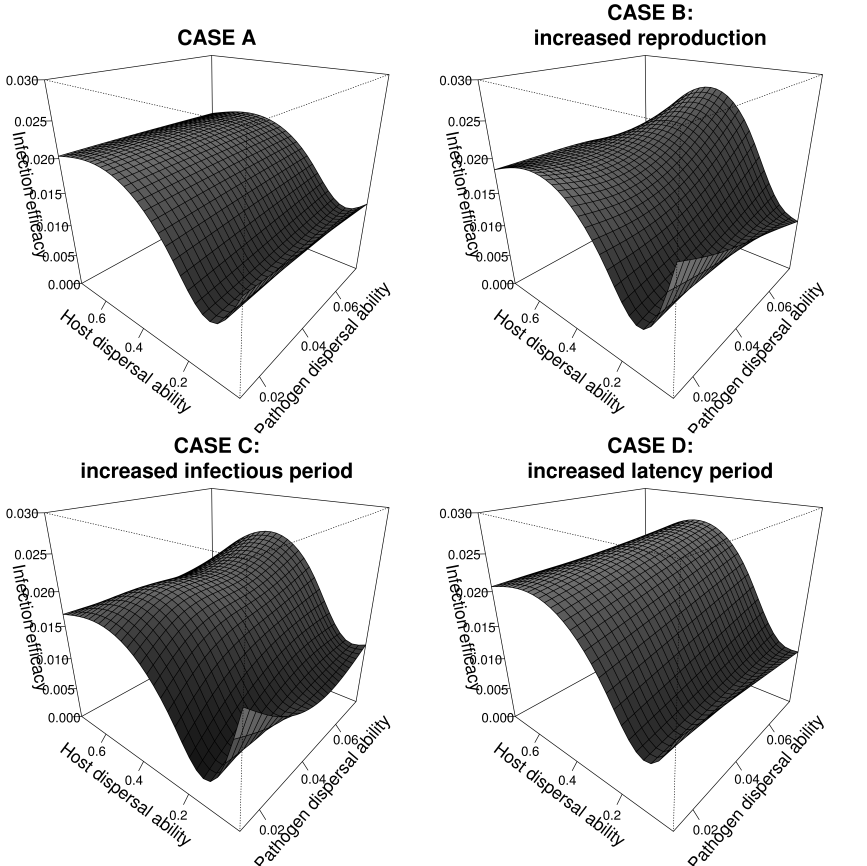


**Figure S10** Efficacy range of the pathogen genetic clusters when 2 genetic clusters coexisted ($\beta=0.9$).


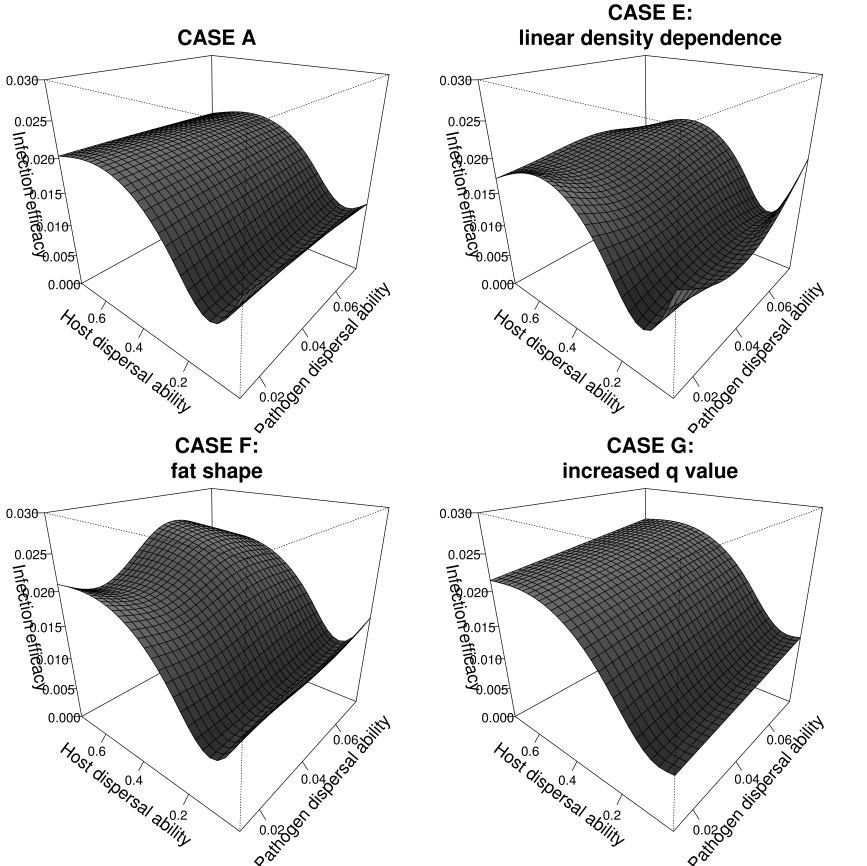


**Figure S11** Efficacy range of the pathogen genetic clusters when 2 genetic clusters coexisted ($\beta=0.9$).


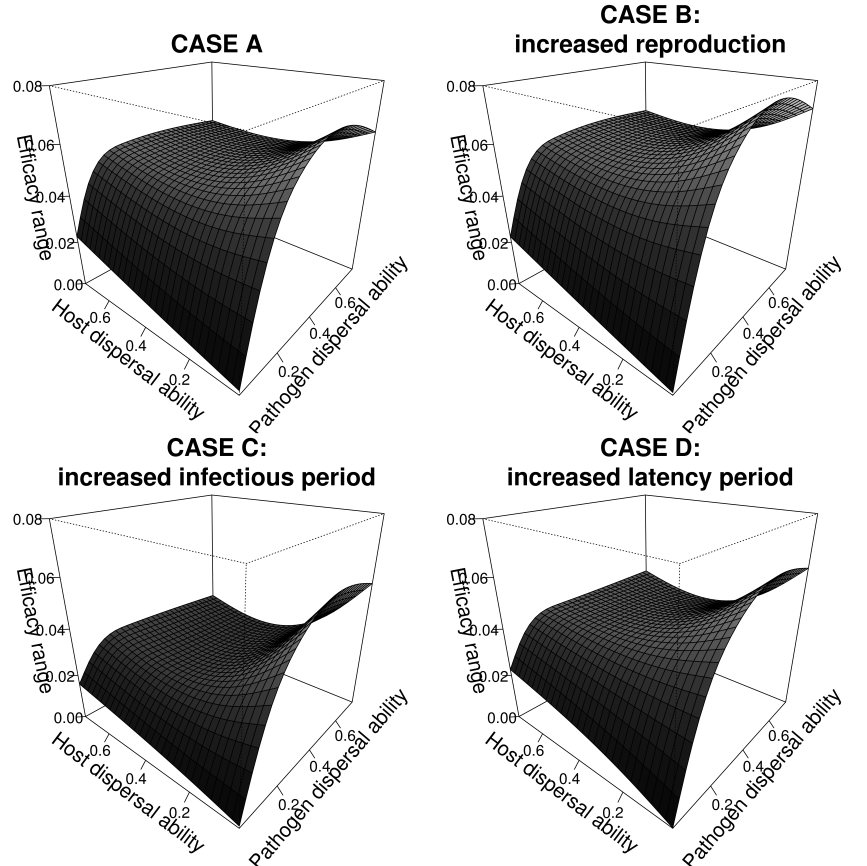


**Figure S12** Efficacy range of the pathogen genetic clusters when only 1 genetic cluster persisted ($\beta=1$).


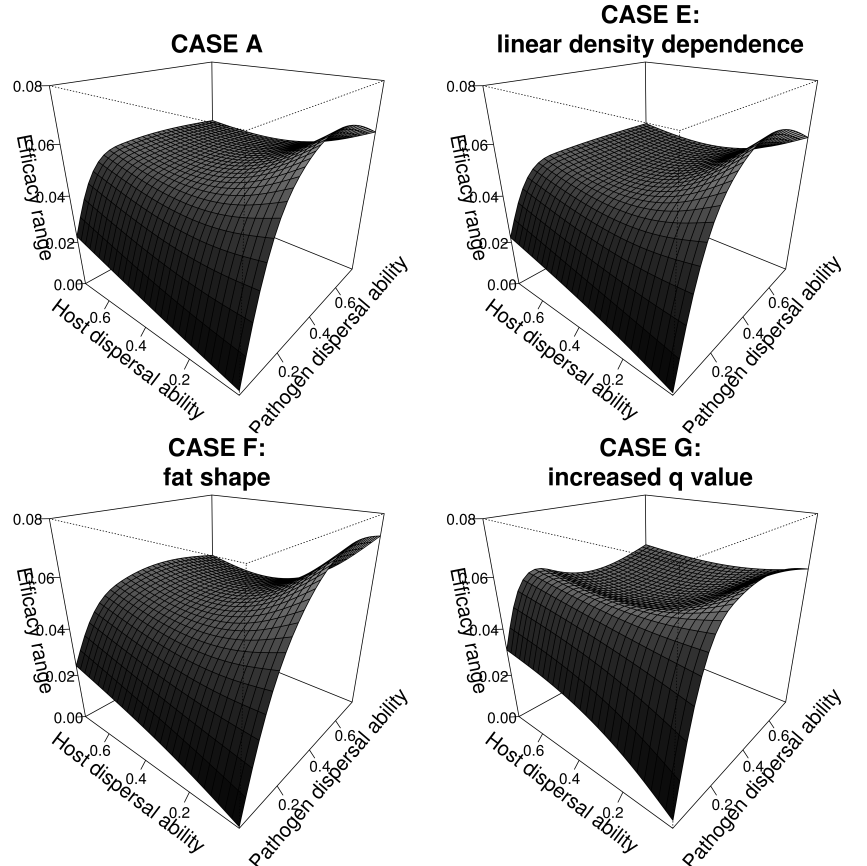


**Figure S13** Efficacy range of the pathogen genetic clusters when only 1 genetic cluster persisted ($\beta=1$).
